# 17β-Estradiol Modulates Cancer Cell–Fibroblast Communication via Autophagy-Mediated Extracellular Vesicle Secretion and Promotes Poor Prognosis in Non-Small Cell Lung Cancer

**DOI:** 10.3390/ijms27167490

**Published:** 2026-08-21

**Authors:** Rosa Vona, Camilla Cittadini, Barbara Ascione, Lucrezia Gambardella, Katia Fecchi, Lucia Bertuccini, Annalisa Tocci, Lorenzo D’Ambrosio, Maria Cristina Gagliardi, Federica Felicetti, Elena Ortona, Paola Nisticò, Anna Maria Mileo, Paola Matarrese

**Affiliations:** 1 Center for Gender-Specific Medicine, Istituto Superiore di Sanità [Italian National Institute of Health], Viale Regina Elena, 299-00161 Rome, Italy; barbara.ascione@iss.it (B.A.); lucrezia.gambardella@iss.it (L.G.); elena.ortona@iss.it (E.O.); 2 Department of Infectious Diseases, Istituto Superiore di Sanità, Viale Regina Elena, 299-00161 Rome, Italy; camilla.cittadini@iss.it (C.C.); katia.fecchi@iss.it (K.F.); mariacristina.gagliardi@iss.it (M.C.G.); 3 Core Facilities, Italian National Institute of Health, Viale Regina Elena, 299-00161 Rome, Italy; lucia.bertuccini@iss.it (L.B.); federica.felicetti@iss.it (F.F.); 4 Tumor Immunology and Immunotherapy Unit, IRCCS Regina Elena National Cancer Institute, Via Elio Chianesi, 53-00144 Rome, Italy; annalisa.tocci@ifo.it (A.T.); lorenzo.dambrosio@ifo.it (L.D.); paola.nistico@ifo.it (P.N.); annamaria.mileo@ifo.it (A.M.M.)

**Keywords:** lung cancer, tumor progression, tumor microenvironment, estrogens, estrogen receptor β

## Abstract

Non-small cell lung cancer (NSCLC) remains a leading cause of cancer-related mortality. Smoking is the primary etiological factor, but growing evidence suggests the involvement of estrogen in its development and progression, although its role remains unclear. This study explores: (i) the effects induced by estrogen, namely, 17β-estradiol (E2), alone or in combination with a mixture of inflammatory cytokines (Mix), in two human NSCLC cell lines, A549 and Calu1, and (ii) whether and how tumor cells can modulate the activation of normal lung fibroblasts. We found that E2 significantly enhances migration, invasion, and epithelial–mesenchymal transition in NSCLC cells, as well as their resistance to cisplatin, particularly in combination with Mix. Pharmacological inhibition of ERβ reversed the E2-induced effects, implicating ERβ in E2-mediated signaling. Furthermore, E2 increased autophagic flux and induced a shift toward secretory autophagy and the release of extracellular vesicles, which activated normal lung fibroblasts, as demonstrated by the increased expression of α-SMA, FAP, PDGFR-β, and PDPN. The clinical relevance of these data was supported by computational analyses revealing an elevated expression of the Mix gene signature, including TGF-β, IL-6, IL-8, CCXL-16, and ERβ, which was associated with shorter overall survival in NSCLC patients. This molecular profile was linked to the elevated expression of secretory autophagy genes and cancer-associated fibroblast markers. Validation in three large clinical cohorts (TCGA-LUNG, OAK and POPLAR) strengthens the clinical relevance of this E2-related pro-tumor axis while suggesting a promising therapeutic avenue for NSCLC patients.

## 1. Introduction

Non-small cell lung cancer (NSCLC) accounts for approximately 85% of all lung cancers and represents a significant global health burden, as it is one of the leading causes of cancer-related mortality worldwide. While smoking remains the predominant risk factor for NSCLC, emerging evidence suggests that hormonal factors, particularly estrogen, may also play a critical role in the pathogenesis and progression of this malignancy.

Estrogen exerts multiple physiological effects by two major receptors, estrogen receptor alpha (ERα) and estrogen receptor beta (ERβ), which belong to the nuclear receptor superfamily and act as ligand-activated transcription factors [1]. These receptors are widely expressed in various tissues, including the lung, where they regulate cell proliferation, differentiation, apoptosis and angiogenesis [2]. The presence of estrogen receptors in lung tissue has led to investigations into the possible involvement of estrogen signaling in lung cancer development [3,4]. Preclinical studies have demonstrated the expression of estrogen receptors in NSCLC cell lines and in patient-derived tumor samples, further implicating estrogen signaling in lung cancer biology [5]. The pleiotropic effects of estrogen on NSCLC pathogenesis are thought to be mediated by various mechanisms, including the promotion of tumor cell proliferation, inhibition of apoptosis, regulation of immune responses and modulation of the tumor microenvironment (TME) [6]. Although the promoting role of 17β-estradiol (E2) in NSCLC is well known [7], literature data on the role of ERs in the initiation, progression and prognosis of NSCLC remain controversial. Some studies report an association between poor prognosis and ERα signaling, while others report on ERβ [8,9,10,11,12,13,14]. The crosstalk between tumor cells and fibroblasts is mediated by specific cytokines and may affect various biological processes that promote cancer progression and resistance to therapy [15].

Literature data highlight the key role played by the autophagic process in tumor progression [16]. In particular, evidence has emerged recently on the role of autophagy in secretory processes [17,18] and endocytic trafficking [19,20]. Indeed, it has been demonstrated that the release of secreted factors through the autophagic process is able to favor an invasive cancer phenotype and promote the immunosuppressive properties of the TME [21,22]. This would highlight that autophagy is not only a lysosomal degradation process but can also play important roles in the extracellular secretion of bioactive molecules, including cytokines. In recent years, extracellular vesicles (EVs) have emerged as critical mediators of intercellular communication within the TME landscape. EVs, including exosomes, microvesicles, and apoptotic bodies, are membrane-bound nanovesicles released by various cell types, including tumor cells and stromal cells, and play multiple roles in cancer progression, including the modulation of the immune response, promotion of angiogenesis and establishment of pre-metastatic niches. Of particular interest are exosomes, a subtype of EVs with a diameter of 30–160 nm that are generated by the endolysosomal pathway and secreted following the fusion of multiple vesicular bodies (MVBs) with the plasma membrane. They play a key role in the regulation of pathophysiological processes and have been implicated in various aspects of NSCLC pathogenesis. Recent studies have provided evidence of crosstalk between the endosomal pathways of exosome biogenesis and autophagy, resulting in the release of exosomes that include autophagic components. To date, specific markers for EV subtypes that allow the identification of their endosomal or plasma membrane origin have not yet emerged. Consequently, it is difficult to assign an EV population to a specific biogenesis pathway; thus, we will refer to our secreted vesicles as small EVs [23].

This work investigates the still undefined role of estrogens, particularly E2, in the crosstalk between CAFs and tumor cells in NSCLC.

Our experimental data indicate that E2 modulates the crosstalk between tumor cells and fibroblasts by increasing extracellular vesicle secretion through autophagy, thereby enhancing cancer cell invasiveness and resistance to cisplatin. Clinically, by exploring public NSCLC datasets, we found that E2 receptors, specific cytokines, and genes related to secretory autophagy stratify NSCLC patients with poor prognosis. Future studies will validate the relevance of estrogens in the development of tailored treatments and in designing novel combination therapies for NSCLC.

## 2. Results

In a recent study, we demonstrated that a combination of specific inflammatory cytokines (i.e., TGF-β, IL-6, IL-8 and CCXL-16) promoted migration, invasion and epithelial–mesenchymal transition (EMT) in the cultured non-small cell lung cancer (NSCLC) cell lines A549 (adenocarcinoma) and Calu1 (epidermoid carcinoma) through activation of the YAP-mediated autophagic process [24].

Therefore, based on the emerging relevance of estrogens in the progression of NSCLC [25], we focused on the effects induced by 17β-estradiol (E2), administered alone or in combination with IL-6, IL-8, TGF-β and CCXL-16 (referred to as the Mix), to mimic the influence of microenvironmental factors on tumor progression. Since E2 produces its effects by interacting with specific receptors present ubiquitously in all tissues regardless of sex, before proceeding to treatments with E2, we analyzed the expression of the estrogen receptors ERα and ERβ in A549 and Calu1 cells. As shown in Supplementary Figure S1, both cell lines expressed a higher amount of ERβ than ERα. Flow cytometry analysis also demonstrated the presence of both receptors on the plasma membrane. With regard to intracellular localization, immunofluorescence microscopy revealed a significant amount of ERα, but not ERβ, even in the nucleus of both cell lines. Nuclear localization was much more marked in Calu1 cells (Supplementary Figure S1).

### 2.1. E2 Promotes Cell Motility and Invasiveness

**Figure 1:**
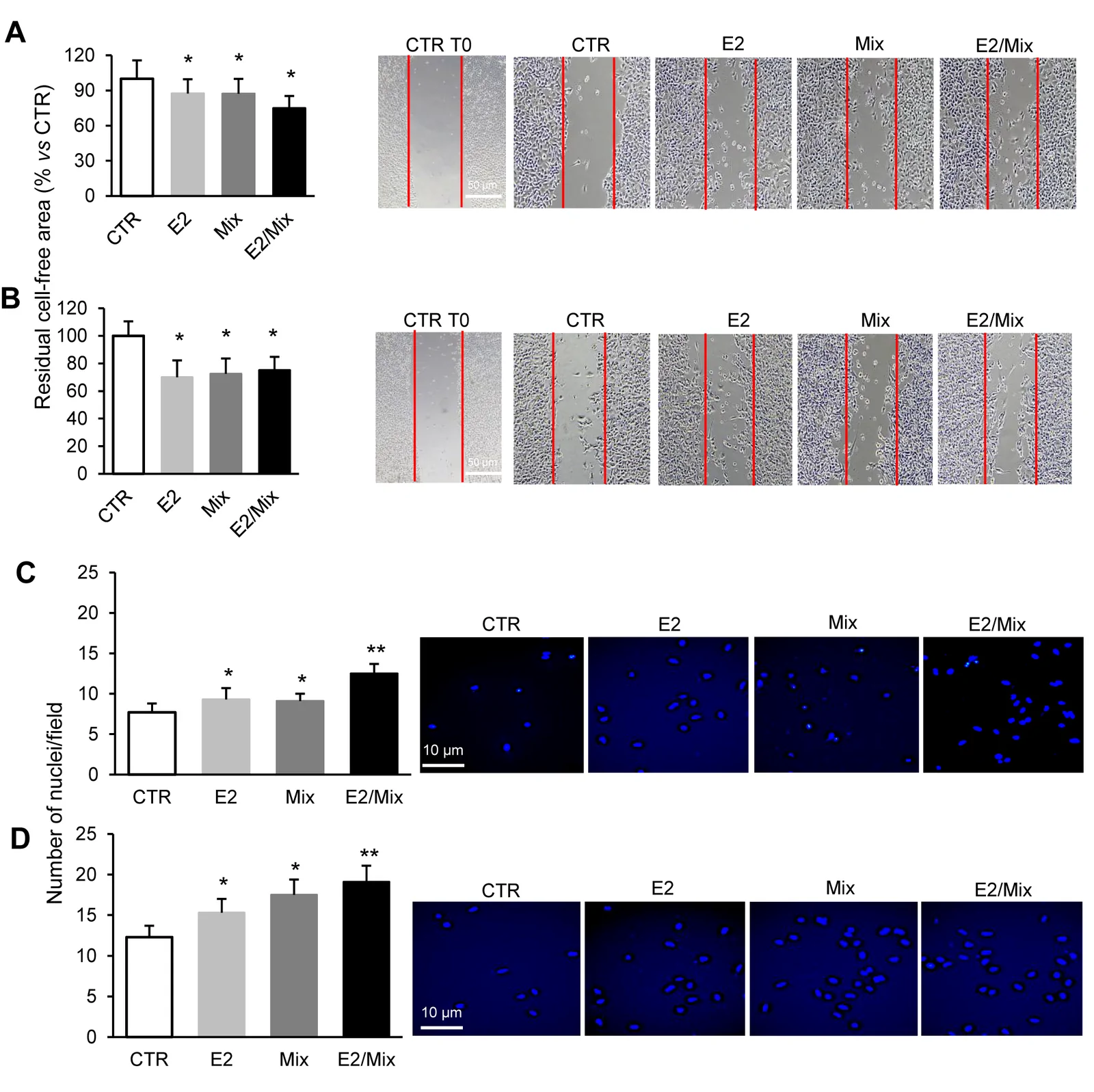
E2 promotes cell motility and invasiveness. Migration assay performed by scratch assay on (**A**) A549 and (**B**) Calu1 cells. Bar graph: Quantification of wound reduction after 48 h in treated cells compared to control cells, set as 100%. Note: Lower cell-free area values indicate greater migration. Phase-contrast micrographs: Representative images of three independent experiments. The invasion assay was performed using Matrigel-coated Transwell culture inserts with 8.0 µm pores containing (**C**) A549 and (**D**) Calu1 cells. Bar graph: Number of cells migrated to the bottom of the filter. Fluorescence micrographs show representative images after Hoechst staining of cells invading the bottom of the filter. One-way ANOVA followed by Bonferroni’s post hoc test was used to determine statistical significance, which was defined as * *p* < 0.05 and ** *p* < 0.01 versus the respective controls.

We evaluated the ability of E2, alone or in combination with the mixture of cytokines (E2/Mix), to interfere with cell motility. A549 and Calu1 cells were brought to confluence and then subjected to a wound as described (in vitro scratch assay; [26]). The treated and untreated cells were then immediately incubated, and the data were collected by photographing the lesions at t 0, 24 and 48 h after lesion induction. Cells treated with E2, Mix or the combination of the two showed a greater motility and a significant increase in their ability to migrate, i.e., to repopulate the wound area, as shown in the representative images and histograms for the A549 (Figure 1A) and Calu1 (Figure 1B) cell lines. In the same vein, A549 and Calu1 cells were then challenged to investigate their invasion capabilities. In A549 cells, we observed an increase of about 20% in the presence of E2 or Mix and of about 60% in the presence of E2/Mix when control cells were considered as 100% (Figure 1C). In the same experimental context, Calu1 control cells showed a greater propensity to invade than A549 cells, in accordance with their greater aggressiveness (white column in Figure 1D compared with the white column in Figure 1C). After treatment with E2, Mix or E2/Mix in Calu1 cells, we observed a significant increase in their invasion capabilities of about 24%, 42% and 55%, respectively, compared to the respective control (Figure 1D). In both cell lines, the E2/Mix combination was more effective than single treatments in promoting invasion (black columns in Figure 1C,D).

As the remodeling of the actin cytoskeleton contributes to increasing the motility and metastatic spread of tumor cells [27], we evaluated the amount of total actin (using a specific antibody, Supplementary Figure S2A), as well as its monomeric (G-actin, using DNAse I, Supplementary Figure S2B) and polymeric (F-actin, using phalloidin, Supplementary Figure S2C) forms. In A549 cells, both the E2 and Mix treatments significantly increased total actin expression (fold increases of approximately 1.6 and 2.1, respectively; Supplementary Figure S2A, left panels). In contrast, the 1.2-fold increase observed in the E2/Mix combination compared to the control did not reach statistical significance. For Calu1 cells, all treatments induced a significant increase in total actin (Supplementary Figure S2A, right panels), with a fold increase of approximately 1.8 (E2), 2.1 (Mix), and 2.3 (E2/Mix) compared to the untreated control. The polymeric form of actin showed a significant increase only in A549 cells treated with Mix and in Calu1 cells treated with E2/Mix. No quantitative changes in the monomeric form of actin were observed in either cell line under any experimental condition. Fluorescence microscopic observations after phalloidin staining highlighted, although to a different extent, an increase in stress fibers in both cell lines subjected to the different treatments, as indicated by the arrows in the representative images (Supplementary Figure S2D).

The increase in cell motility and invasiveness observed following the treatments was not associated with a parallel increase in proliferation, as neither E2 nor Mix nor their combination produced any significant effect on the cell proliferation capacities of A549 and Calu1 cells (Supplementary Figure S3).

**Figure 2:**
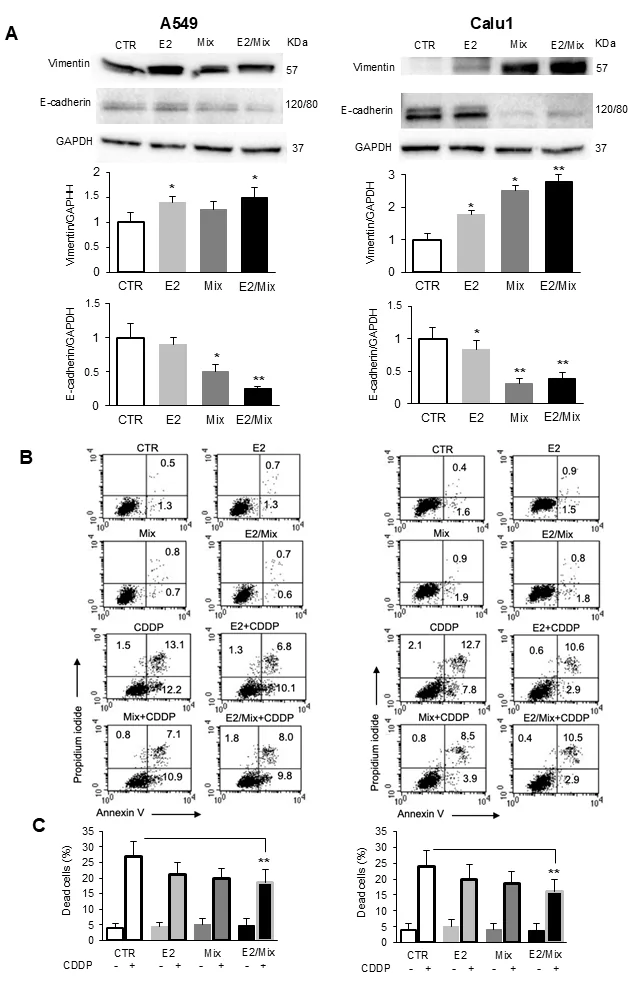
E2 induces EMT and reduces CDDP toxicity. (**A**) Analyses of EMT in A549 and Calu1 cells untreated (CTR) or treated as indicated. Representative western blot analysis of Vimentin, E-cadherin, and GAPDH used as loading controls. Bar graphs and lower panels show densitometry analyses of E-cadherin and Vimentin normalized to GAPDH, presented as the mean ± SD of three independent experiments. (**B**) Cell death analyses performed in A549 and Calu1 cells untreated or treated for 24 h as indicated. Upper panels: biparametric flow cytometry analysis of apoptosis. Results obtained in a representative experiment are shown. Numbers represent the percentage of apoptotic cells: annexin V single-positive (early apoptosis, bottom right quadrant, black columns), annexin V/propidium iodide double-positive (late apoptosis, upper right quadrant), or propidium iodide single-positive (necrotic cells, upper left quadrant). (**C**)The bar graphs represent the mean ± SD of four independent experiments. One-way ANOVA followed by Bonferroni’s post hoc test was used to determine statistical significance, which was defined as * *p* < 0.05 and ** *p* < 0.01 versus the respective controls.

Cell invasiveness during cancer progression is critically linked to the acquisition of EMT [28]; therefore, we aimed to evaluate the changes in the epithelial vs. mesenchymal cell phenotype of NSCLC cells treated or not with E2, Mix or both. We quantified Vimentin and E-cadherin (mesenchymal and epithelial marker, respectively) by western blot analysis of A549 and Calu1 cells (Figure 2A). In agreement with the functional data, we found that all treatments induced a significant increase in Vimentin and a substantial decrease in E-cadherin expression in both cell lines, thus suggesting that even exposure to physiological concentrations of E2 alone can induce activation of the EMT process.

It is well known that EMT is a process that can be associated with drug resistance [29]. Thus, we evaluated if E2 and Mix, alone or in combination, affected cell susceptibility to cisplatin (CDDP). To this aim, after treatment with E2, Mix or their combination, cells were challenged with CDDP for 48 h, and death was assessed by annexin V/propidium iodide staining (Figure 2B). As shown in the dot plot panels, which report data from a representative experiment, and in the bar graphs showing the results obtained from four independent experiments, treatment with E2 or Mix was able to reduce the cytotoxic effects of CDDP in both A549 and Calu1 cells (Figure 2C). This reduction was even more evident in cells treated with the E2/Mix combination.

### 2.2. E2 Induces Its Effects via ERβ

ERβ is the most abundant receptor found in NSCLC, unlike ERα, which is weakly expressed. Additionally, the biological effects of estrogen in the lung seem to be essentially mediated by ERβ [5,12].

**Figure 3:**
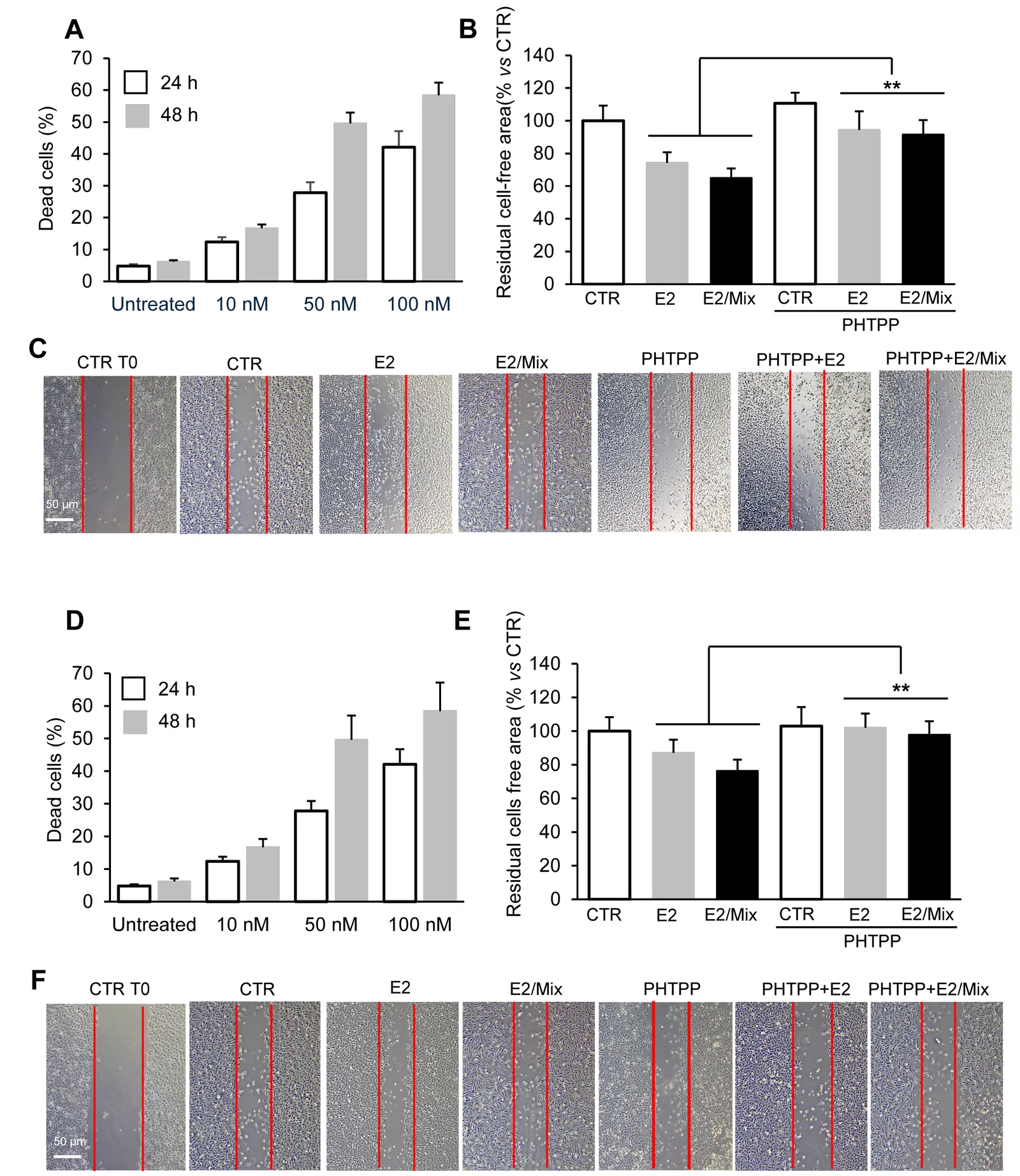
E2 induces its effects via ERβ. (**A**–**C**) A549 cells and (**D**–**F**) Calu1 cells. (**A**,**D**) Bar graphs report the percentage of dead cells after treatment with the ERβ inhibitor PHTPP at the indicated concentrations obtained by flow cytometry after Calcein-AM cell staining. (**B**,**E**) Bar graphs show the quantification of cell migration assessed by scratch assay. The x-axis represents the wound reduction at 48 h in treated cells compared to control cells, which are referred to as 100%, where lower values indicate greater migration. The bar graphs represent the mean ± SD of three independent experiments. One-way ANOVA followed by Bonferroni’s post hoc test for multiple comparisons was used to determine statistical significance, which was defined as ** *p* < 0.01 versus the respective controls. (**C**,**F**) Phase-contrast micrographs show representative images from the scratch test after 48 h. The T0 value is also shown for the CTR alone, since the seeding conditions and initial wound were identical for all treatments.

To verify whether the E2-induced effects were due to the activation of ERβ, ERα or both, we treated cells with PHTPP, a selective ERβ antagonist, or with MPP, a selective ERα antagonist, before adding E2 alone or in combination with Mix. First, we assessed the toxicity of PHTPP (Figure 3A,D refer to A549 and Calu1 cells, respectively) and MPP (data not shown) through a dose–response curve, selecting the concentration of 10 nM for subsequent functional experiments. As a functional test, we evaluated the ability of PHTPP and MPP to inhibit cell migration induced by E2 and E2/Mix. As shown in Figure 3B,E, and in the representative phase-contrast microscopy images shown in Figure 3C,F, PHTPP but not MPP (data not shown) effectively counteracted E2-induced cell migration, alone or in combination with Mix, i.e., it slowed down the repopulation of the experimentally induced wound area. These data strongly suggest the exclusive involvement of ERβ in E2-induced tumor promotion.

### 2.3. E2 Activates EGFR/AKT/ERK Axis

Although ERβ stimulation appears to trigger rapid signaling in collaboration with various kinase cascades, including EGFR and its downstream effectors [27,30], the crosstalk between the ERβ/EGFR pathways has not been explored in lung cancer.

**Figure 4:**
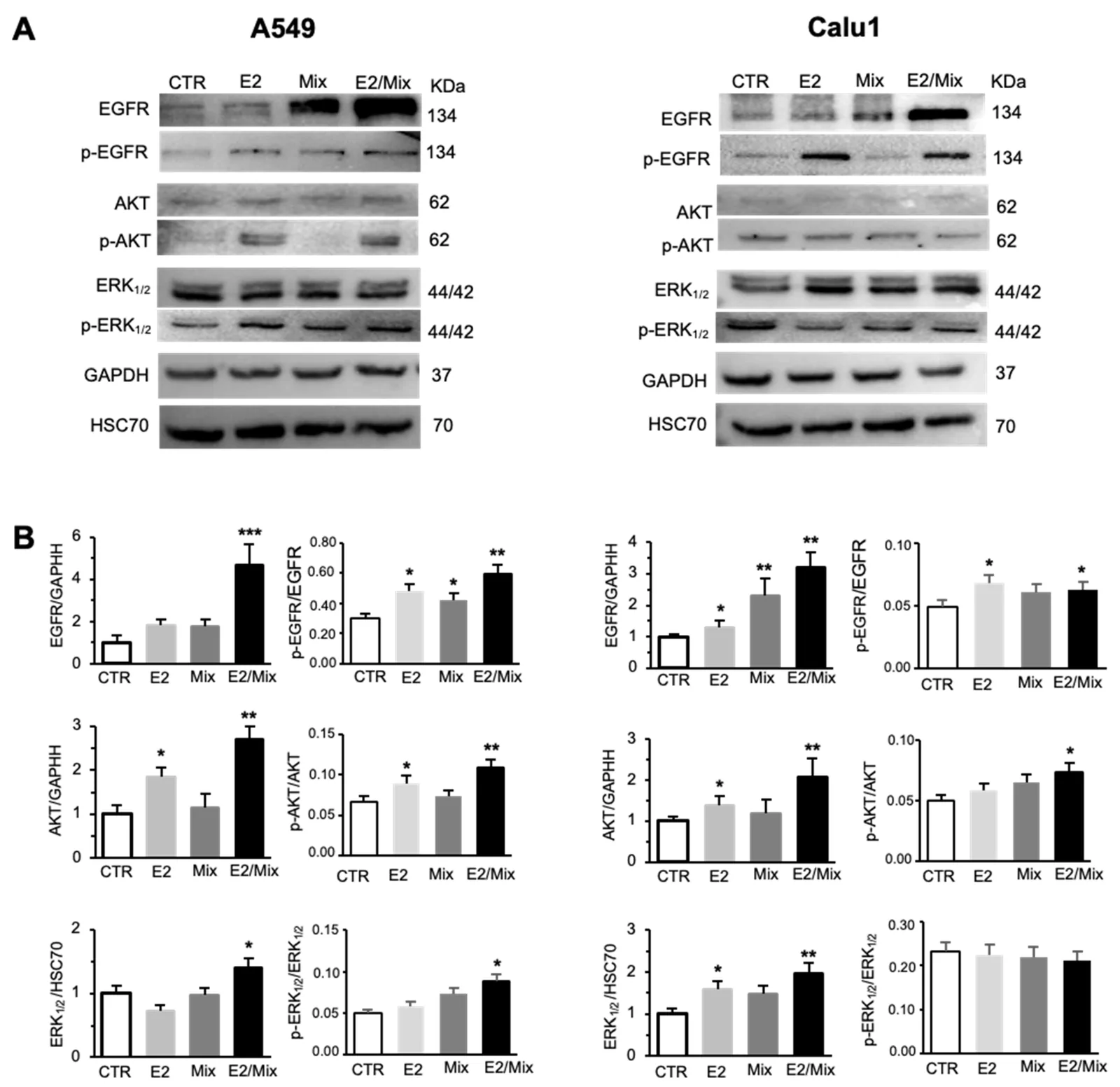
E2 activates EGFR/AKT/ERK axis. (**A**) Western blot analyses of EGFR, AKT, and ERK and their phosphorylated forms in A549 and Calu1 cells that were either untreated (CTR) or treated as indicated. (**B**) The bar graphs show the densitometry analysis of the aforementioned proteins, normalized to GAPDH or HSC70, from three independent experiments. * *p* < 0.05, ** *p* < 0.01 and *** *p* ≤ 0.001 vs. CTR by one-way ANOVA followed by Bonferroni’s post hoc test for multiple comparisons.

To investigate the signaling triggered by ERβ activation in our experimental model, the expression levels of EGFR, AKT, and ERK1/2, including their phosphorylated forms p-EGFR, p-ERK1/2, and p-AKT, were evaluated (Figure 4). Treatment with E2 and Mix, alone or in combination, resulted in increased levels of EGFR and AKT and their phosphorylated forms, p-EGFR and p-AKT, in both cell lines. In Calu1 cells, however, this increase was significant only for p-AKT. As for ERK, in A549 cells, a change in its levels was observed only following the combined treatment, while in Calu1 cells, only E2 treatment induced a significant increase in this protein. p-ERK levels increased in A549 cells after E2 treatment, while in Calu1 cells, the levels remained unchanged across all the experimental conditions.

### 2.4. E2 Activates NF-κB Signaling

**Figure 5:**
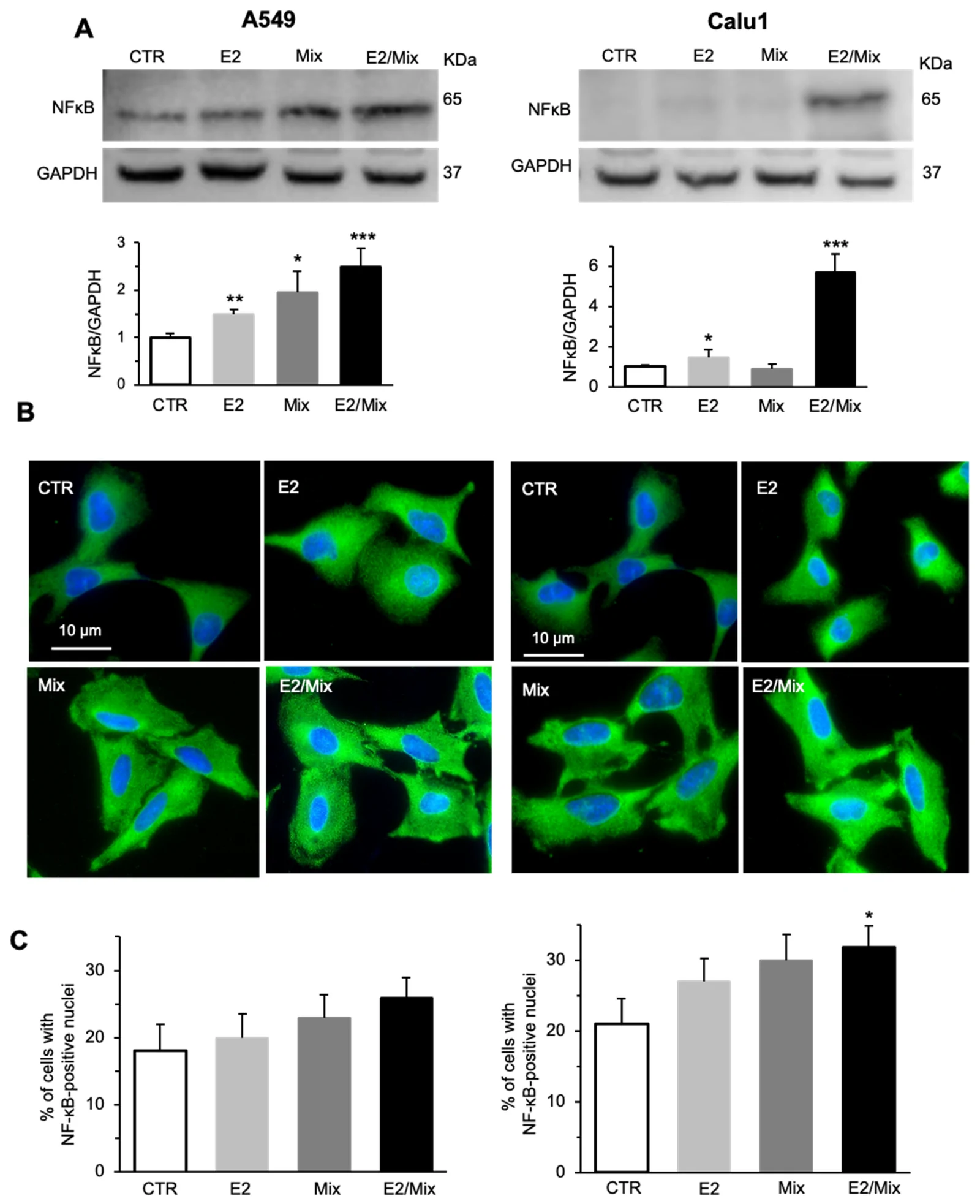
E2 activates NF-κB signaling. (**A**) Western blot analysis of NF-κB in A549 and C alu1 cells from a representative experiment. The bottom bar graphs show densitometry analyses of NF-κB normalized to GAPDH from three independent experiments and reported as the mean ± SD of the three independent experiments. (**B**) The immunofluorescence micrographs show representative images of untreated cells (CTR) or cells treated as indicated and stained for NF-κB. (**C**) Bar graphs report the percentage of cells in which NF-κB was re-localized to the nucleus, calculated by the morphometric analysis performed as specified in Section 4. * *p* < 0.05, ** *p* < 0.01 and *** *p* ≤ 0.001 vs. CTR by one-way ANOVA followed by Bonferroni’s post hoc test for multiple comparisons.

Since it has been reported that EGFR can trigger the degradation of IκBα, thereby activating NF-κB to promote cell survival, we also analyzed the amount and intracellular localization of this transcription factor (Figure 5). Interestingly, NF-κB levels increased following E2 administration. In particular, a significant increase in this protein was observed in A549 cells (Figure 5A) after treatment with E2, alone or in combination with the cytokine mix, while in Calu1 cells, the NF-κB increase became significant only following the E2/Mix combination treatment. Immunofluorescence observations (Figure 5B) followed by a morphometric analysis (Figure 5C) also revealed the re-localization of NF-κB into the nucleus in both cell lines treated with E2, alone or in combination with Mix.

### 2.5. E2 Favors Both Conventional and Non-Conventional Autophagy

**Figure 6:**
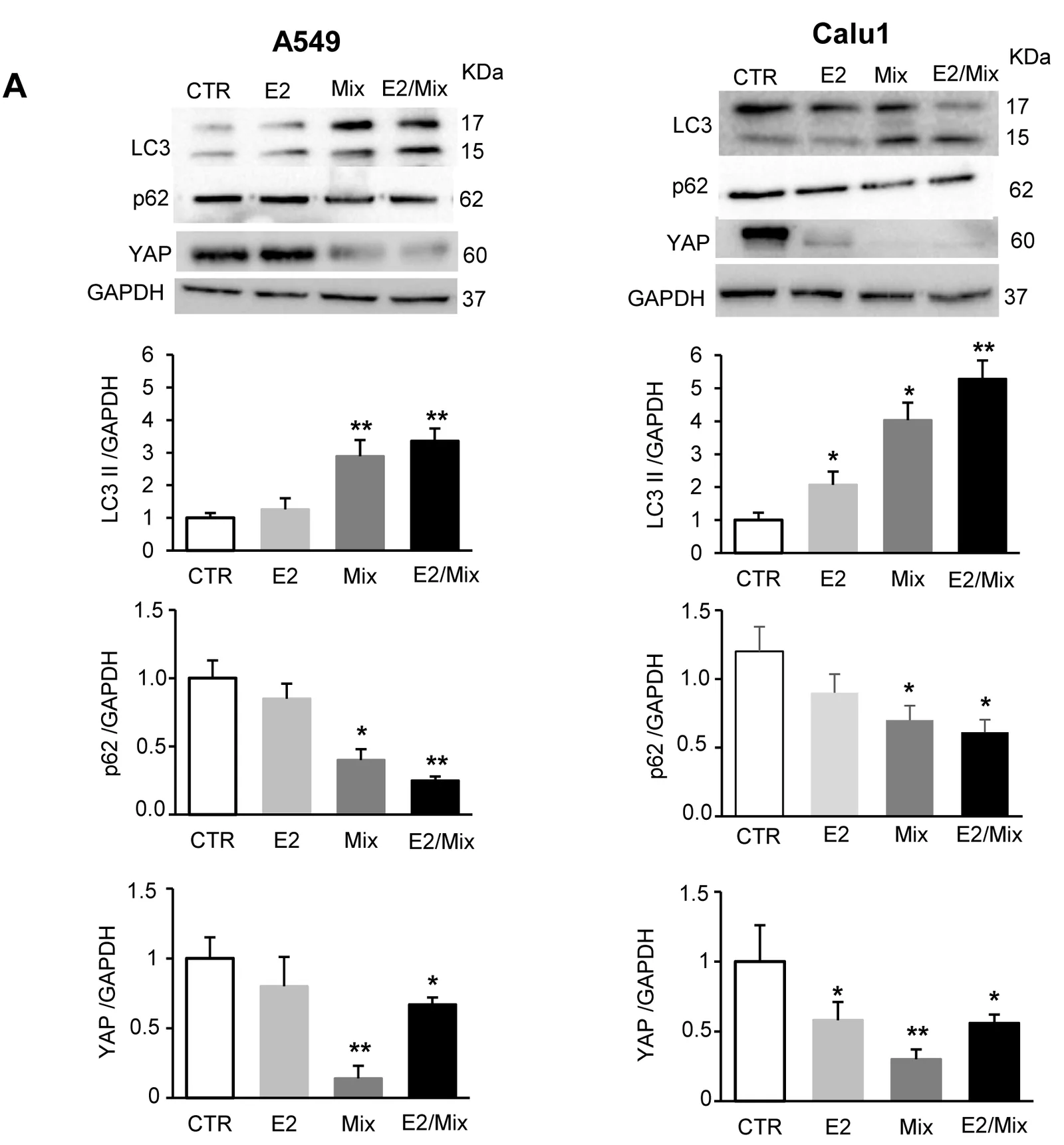
E2 favors conventional autophagy. Upper panels: western blot analysis of autophagy by monitoring LC3, p62, and YAP, using GAPDH as a loading control, in A549 and Calu1 cells untreated (CTR) or treated for 24 h as indicated. Bar graphs show densitometric analyses of each protein normalized to GAPDH. Data are expressed as mean ± SD of three independent experiments. * *p* < 0.05 and ** *p* ≤ 0.001 vs. CTR by one-way ANOVA followed by Bonferroni’s post hoc test to determine statistical significance.

Autophagy plays an important role in resistance to CDDP treatment in lung cancer [31]. Furthermore, it is known that EGFR-activated signaling pathways can modulate autophagy in a pro- or anti-tumor ways [32]. We therefore wanted to verify whether autophagy was also involved in the effects observed following E2 treatment. As shown in Figure 6, western blotting analysis, followed by densitometric evaluation, of LC3I/II and p62 revealed a clear induction of autophagy in response to E2 in both NSCLC cell lines, confirming previously published effects of the combination of inflammatory cytokines. Indeed, treatment with E2 and Mix, alone or in combination, significantly reduced p62 while simultaneously increasing the amount of LC3II in both A549 and Calu1 cells (Figure 6). Additionally, the level of YAP appeared to be significantly reduced, confirming that YAP is also a substrate of autophagy and is degraded in autolysosomes [24,33]. Consistent with the biochemical data, fluorescence microscopy also revealed a significant increase in cytoplasmic LC3-II puncta after E2 or Mix treatment in both A549 (Supplementary Figure S4) and Calu1 (Supplementary Figure S5) cells.

**Figure 7:**
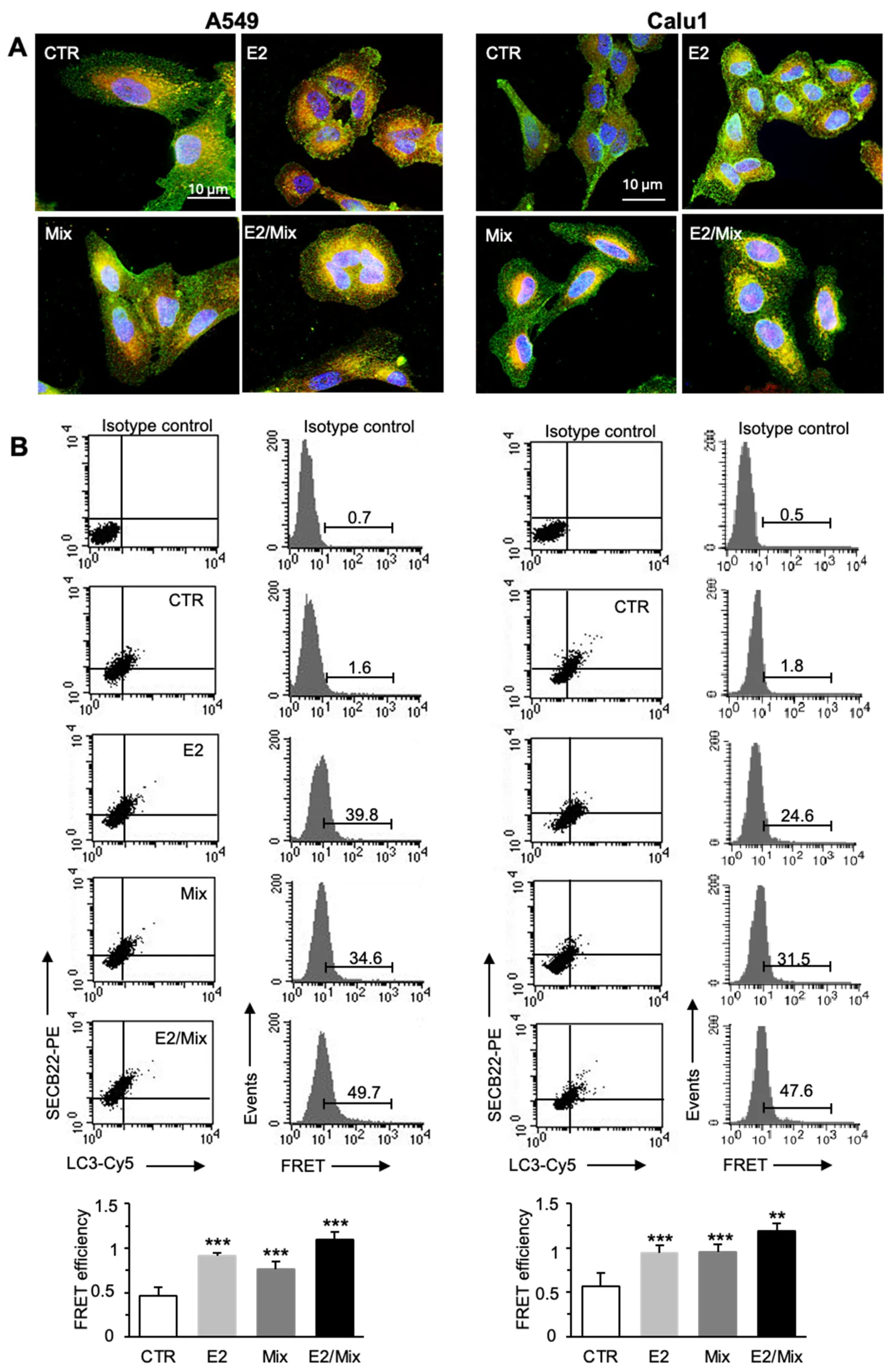
E2 favors non-conventional autophagy. (**A**) Immunofluorescence micrographs show representative images of A549 cells and Calu1 cells stained for LC3 (green) and SEC22B (red) and counterstained with Hoechst (blue). Yellow areas indicate colocalization of LC3 and SEC22B. (**B**) Quantitative evaluation of LC3/SEC22B association by FRET technique as revealed by flow cytometry analysis. Numbers in flow cytometry curve panels indicate the percentage of FL3-positive events (FRET channel) obtained in one experiment that is representative of three experiments. The bar graphs show the FRET efficiency—as an index of the association between LC3 and SEC22B—calculated according to the Riemann algorithm. Bar graphs represent the mean ± SD of three independent experiments. Statistical analyses were performed using one-way ANOVA followed by Bonferroni’s post hoc test for multiple comparisons. ** *p* < 0.01, and *** *p* < 0.001 vs. CTR.

Immunofluorescence images drew attention to the presence of LC3 vesicles at the cytoplasmic periphery, close to the plasma membrane, in all the observed samples but was more evident in the cells treated with E2. Specific markers present on the membrane of autophagosomes allow us to discriminate between degradative and secretory autophagosomes. Both the degradative and secretory pathways are labeled with LC3, but while its association with STX17 directs the autophagosome towards fusion with the lysosome (degradative), the molecular association of LC3 with SEC22B directs the autophagosome towards its own secretion [18,34]. According to this, fluorescence microscopy analysis showed that the co-localization of SEC22B with LC3 (yellow areas) observed in untreated cells significantly increased 24 h after E2, Mix or E2/Mix exposure both in A549 and Calu1 cells (Figure 7A). In representative immunofluorescence images of cells labeled for LC3 (green) and SEC22B (red), large areas of co-localization of these two proteins (yellow) can be observed after all treatments, but especially in cells treated with the E2/Mix combination. Quantitative FRET analysis (Figure 7B) also demonstrated an increased LC3/SEC22B association after the administration of E2, Mix, or their combination either in A549 or in Calu1 cells (bottom panels of Figure 7B).

### 2.6. E2 Induces Secretory Vesicles via Non-Conventional Autophagy

Intercellular communication in the TME is orchestrated by secretory autophagy through the release of different molecules, including cytokines. These molecules, secreted by tumor and stromal cells, e.g., CAFs, can favor the establishment of an immunosuppressive microenvironment, and/or promote tumor growth, metastasis and drug resistance [35].

**Figure 8:**
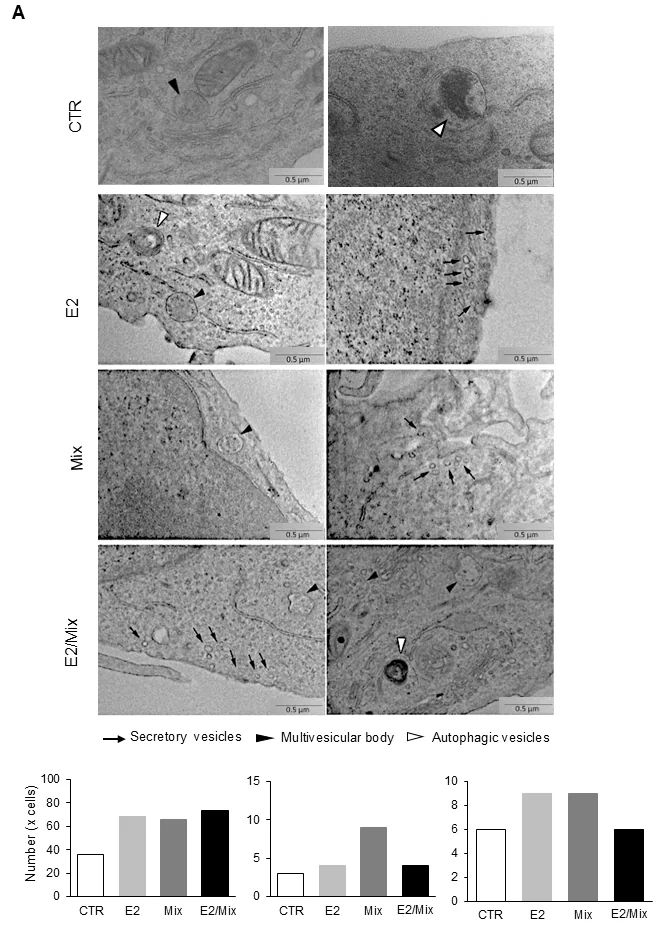

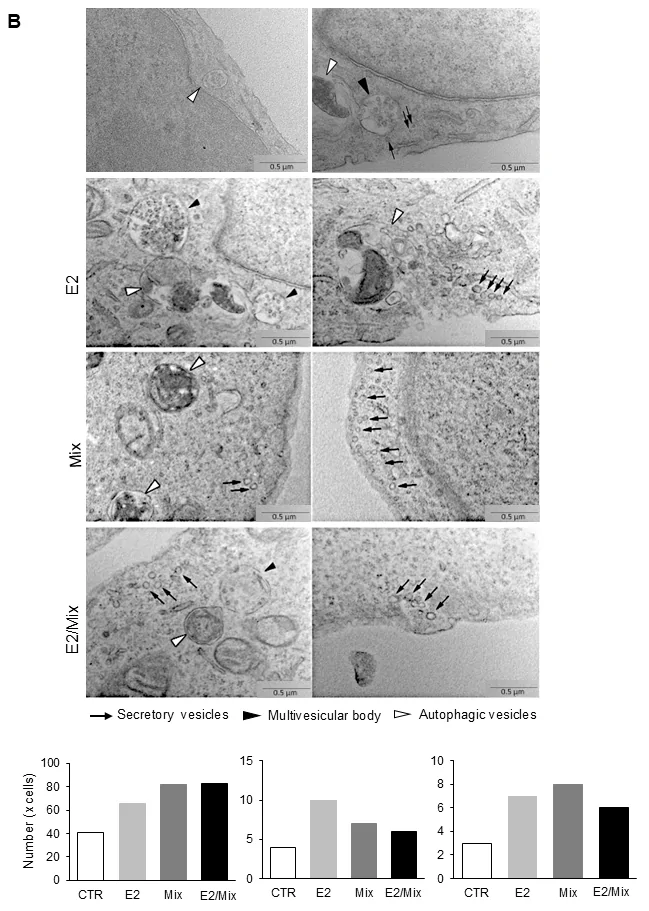
E2 induces secretory vesicles. Transmission electron microscopy analysis of (**A**) A549 cells and (**B**) Calu1 cells treated as indicated (for untreated cells, see Supplementary Figure S3). Numerous small vesicles morphologically characterized by an electron-translucent core can be detected in the cytoplasm in proximity to the plasma membrane (arrows) that can be interpreted as vesicles destined to be secreted. The empty arrowhead indicates an autophagic vacuole, while the black arrowhead indicates multivesicular bodies. Bar graphs report the results of the morphometric analysis performed as specified in Section 4.

Ultrastructural analysis conducted by transmission electron microscopy (TEM, Figure 8) revealed the presence of numerous secretory vesicles often localized close to the plasma membrane (arrows), some autophagic vesicles (white arrowheads) and multivesicular bodies (black arrowheads) in cells treated with E2, Mix and E2/Mix both in A549 (Figure 8A) and Calu1 (Figure 8B) cells, which suggests, according to the above immunofluorescence and FRET data, an ongoing vesicular secretion.

### 2.7. E2-Induced Extracellular Vesicles Activate Primary Lung Fibroblast Conversion into CAFs

Based on these observations, we isolated by sequential ultracentrifugation small EVs from the conditioned medium of A549 and Calu1 cells left untreated (CTR) or treated with E2, Mix or E2/Mix.

**Figure 9:**
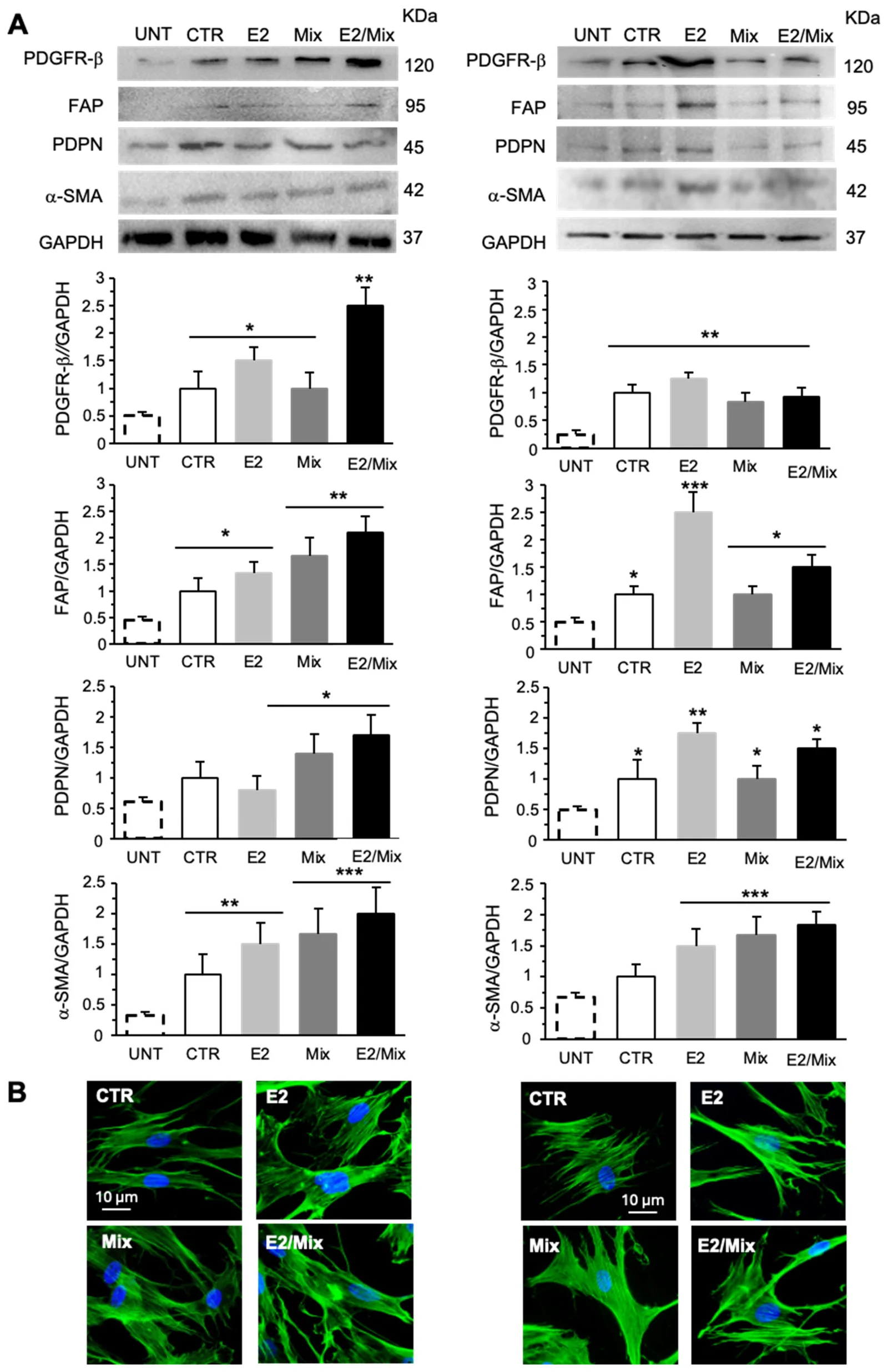
E2-induced extracellular vesicles activate primary lung fibroblast conversion into CAFs. Upper panels: western blot analysis of CAF transformation by monitoring PDGFR-β, FAP, PDPN, and α-SMA in the MRC-5 normal lung fibroblasts untreated (UNT) or incubated for 24 h with the supernatants of (**A**) A549 cells or (**B**) Calu1 cells left untreated (CTR) or treated with E2, Mix or E2/Mix. GAPDH was used as a loading control. Bar graphs show densitometric analyses of each protein normalized to GAPDH as the mean ± SD of three independent experiments. Statistical analyses were performed using one-way ANOVA followed by Bonferroni’s post hoc test. * *p* ≤ 0.05, ** *p* ≤ 0.01, and *** *p* ≤ 0.001 vs. CTR. The immunofluorescence micrographs at the bottom show representative images of MRC-5 normal lung fibroblasts untreated (CTR) or treated as indicated and stained for α-SMA (green) and counterstained with Hoechst (blue).

We first studied the size distribution and relative abundance of released EVs using nanoparticle tracking analysis (NTA). The results showed that EVs isolated from different samples were within the expected size range (30–160 nm) for exosomes. In addition, the protein expression of the exosome markers TSG101, Hsp90 and Alix was confirmed by western blotting analysis (Supplementary Figure S6). EVs isolated from the culture media of A549 and Calu1 cells (untreated or treated with E2, Mix or both) were then administered for 24 h to MRC-5 primary lung fibroblasts. Specific markers of CAFs such as Platelet-Derived Growth Factor Receptor-beta (PDGFR-β), Fibroblast-Activated Protein (FAP), Podoplanin (PDPN), and Alpha smooth muscle actin (α-SMA) were then analyzed by western blotting in the normal MRC-5 lung fibroblasts (Figure 9A). Interestingly, after 24 h of incubation with EVs obtained from A549 and Calu1 cells, both control and primary-treated MRC-5 fibroblasts showed a significant increase in all CAF markers, although at different levels, as clearly demonstrated by densitometric analysis. Note that EVs isolated from A549 cells treated with the E2/Mix combination induced the highest increase in fibroblast activation markers compared to both control and singly treated cells. As for vesicles isolated from Calu1 cells, E2 induced a greater increase in the expression of PDGFR-β, FAP and PDPN than the E2/Mix combination.

In Figure 9B, representative immunofluorescence images of MRC-5 cells labeled for α-SMA after incubation with EVs derived from tumor cells untreated (CTR) or after different treatments are shown. These results indicate that EVs produced by tumor cells after treatment with E2, Mix or their combination induce the activation of primary fibroblast conversion into CAFs.

### 2.8. Multi-Cohort Analysis Validates Estrogen’s Role in the Secretory Autophagy Process and Fibroblast Activation and the Correlation with NSCLC Patient Survival

**Figure 10:**
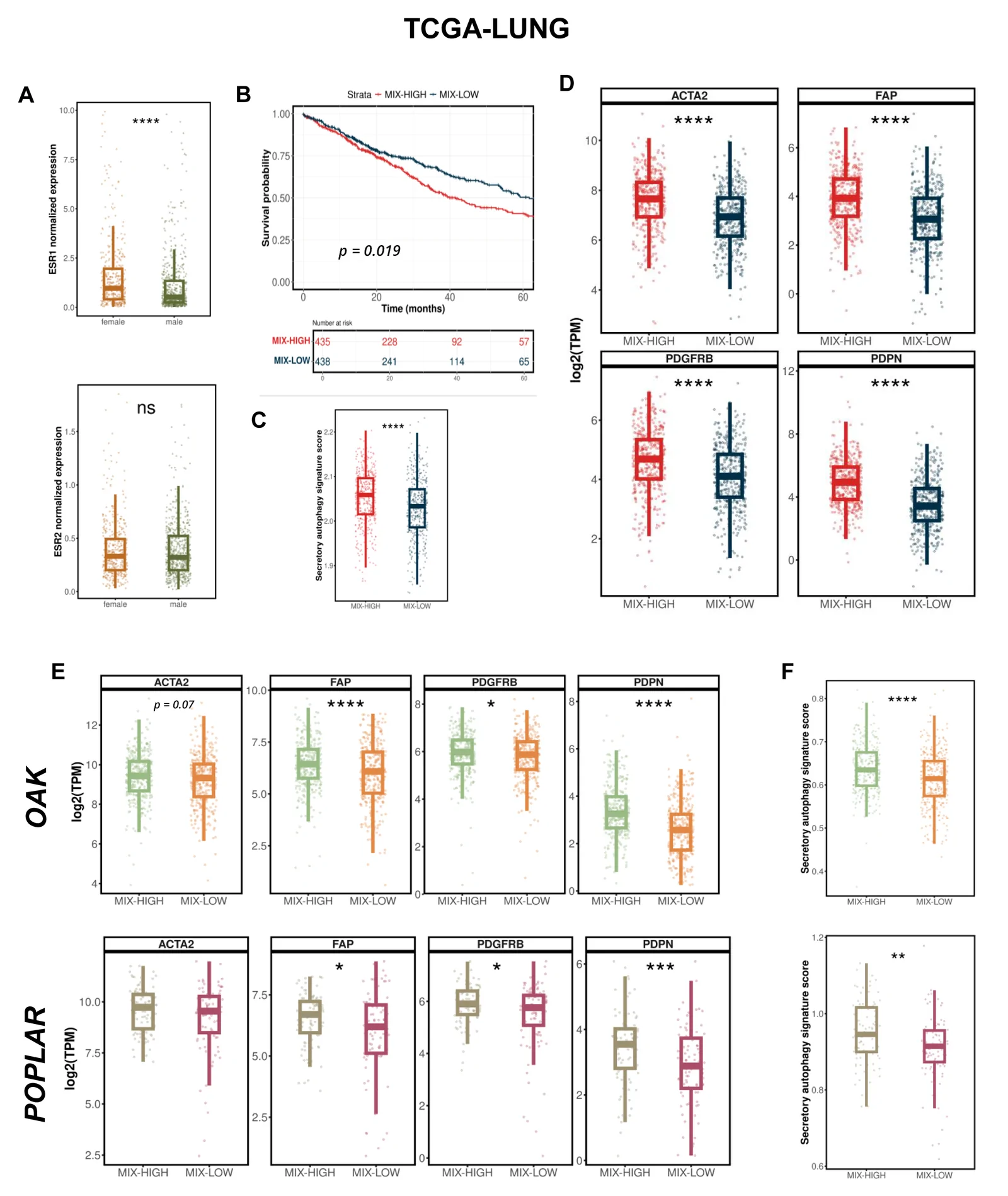
(**A**) Boxplots showing the expression levels of ESR1 (**top**) and ESR2 (**bottom**) genes in the TCGA-LUNG cohort females vs. males. The comparison did not show expression level differences for ESR2 gene. (**B**) Kaplan–Meier curve showing overall survival (OS) for TCGA-LUNG cohort patients stratified into MIX-HIGH and MIX-LOW groups based on the score of a gene signature that comprises *TGFB1*, *IL-8*, *IL-6*, *CCXL-16* and *ESR2* genes. The MIX-HIGH group exhibits significantly worse OS compared to the MIX-LOW group (*p* = 0.019, log-rank test). (**C**) Boxplot displaying the distribution of the score of a secretory autophagy signature (ATG7, ATG9A, MAP1LC3B, TRIM16, RAB8A and SEC22B genes) in the MIX-HIGH and MIX-LOW groups within the TCGA-LUNG cohort (ssGSEA method). The signature score is significantly higher in the MIX-HIGH group (****, Wilcoxon rank-sum test). (**D**,**E**) Boxplots showing the expression levels of four CAF-related genes, each significantly higher in the MIX-HIGH group compared to the MIX-LOW group in the (**D**) TCGA-LUNG (****, Wilcoxon rank-sum test), (**E**, **top**) OAK (****, Wilcoxon rank-sum test), and (**E**, **bottom**) POPLAR (****, Wilcoxon rank-sum test) cohorts. (**F**) Boxplots illustrating the distribution of a secretory autophagy signature score (ssGSEA method) in the MIX-HIGH and MIX-LOW groups in the OAK (**top**) and POPLAR (**bottom**) cohorts. The signature score is significantly higher in the MIX-HIGH group in both cohorts (****, Wilcoxon rank-sum test). For boxplots, the center line indicates the median, the box represents the interquartile range (IQR), and whiskers extend to 1.5 times the IQR from the box edges. Statistical significance is denoted as: * *p* < 0.05, ** *p* < 0.01, *** *p* < 0.001, and **** *p* < 0.0001.

To further validate our in vitro findings regarding autophagy and CAFs, and to translate them into the clinical setting, we performed computational analyses on publicly available datasets. Firstly, to identify a suitable proxy for E2 expression, we assessed the expression levels of the estrogen receptors *ESR1* and *ESR2* in the TCGA-LUNG cohort. Given the known sex-specific differences in estrogen secretion [36], we compared *ESR1/2* gene expression between male and female NSCLC patients. ESR1 expression was significantly higher in females compared to males, while *ESR2* showed no significant sex-specific differences (Figure 10A). Therefore, we selected *ESR2* as a proxy for E2 and included both male and female specimens in subsequent analyses.

Next, we constructed a gene signature (termed “MIX”) comprising *TGFB1*, *IL-8*, *IL-6, CCXL-16*, and *ESR2*. Stratifying TCGA-LUNG patients by the median expression of the MIX signature score revealed a significant survival advantage in patients with low expression levels (MIX-LOW) (Figure 10B). To exclude the possibility that this association was driven solely by ESR2 expression, we repeated the stratification using ESR2 alone or the four cytokine genes alone; none reached statistical significance individually (Supplementary Figure S7, Supplementary Table S1), supporting a genuine contribution of the combined MIX signature rather than of ESR2 in isolation. Furthermore, MIX-HIGH patients exhibited a higher secretory autophagy gene signature score compared to the MIX-LOW group (Figure 10C), aligning with our in vitro observations on the role of E2 and MIX genes in promoting secretory autophagy. Consistent with our experimental results, four key CAF activation marker genes, namely, *ACTA2*, *FAP*, *PDGFRB*, and *PDPN*, were more highly expressed in the MIX-HIGH group than in the MIX-LOW group, supporting our finding that EVs induced by E2 and Mix proteins favor fibroblast activation (Figure 10D).

To confirm these findings in other independent cohorts, we analyzed pre-treatment RNA-Seq profiles from the OAK (*n* = 700) and POPLAR (*n* = 193) clinical trials. Applying the same stratification strategy, we observed higher expressions of *FAP*, *PDGFR-β* and *PDPN* in the MIX-HIGH group compared to the MIX-LOW group in both datasets (Figure 10E). Although *ACTA2* showed no significant differences, a clear trend towards higher expression in the MIX-HIGH group was noted in the OAK cohort (Figure 10E). Importantly, both external datasets also demonstrated a higher secretory autophagy signature score in the MIX-HIGH group, further corroborating the evidence from the TCGA-LUNG cohort (Figure 10F).

Collectively, our integrated computational and in vitro data consistently demonstrate that estrogen-mediated pathways support secretory autophagy in tumor cells and CAF activation, ultimately contributing to unfavorable patient survival.

## 3. Discussion

Despite advances in cancer treatments, including immunotherapy and targeted therapies, the overall five-year survival rate for patients with NSCLC remains poor [37]. This highlights the urgent need to identify novel therapeutic targets to improve clinical outcomes in NSCLC.

The TME, both the cellular and non-cellular components, drives tumor cell reprogramming towards more aggressive phenotypes [38]. Among the fundamental cellular components of the TME are CAFs, which interact through soluble factors, such as cytokines, to influence tumor fate.

In a previous study, we demonstrated that inflammatory cytokines (i.e., TGF-β, IL-6, IL-8 and CCXL-16) promoted migration, invasion and EMT in NSCLC cells via YAP-mediated autophagic process [24]. Herein, we explored the role of estrogens, specifically E2, in modulating the tumor–CAF interaction. Our results show that in NSCLC cells, treatment with E2, alone or in combination with a mixture of the above cytokines, significantly increased cell motility and invasion properties by a readjustment of cytoskeletal dynamics [39]. Following treatments with Mix and/or E2,we observed an increase in polymeric actin and stress fibers, particularly in Calu1 cells, which was associated with the increase in vimentin and decrease in E-cadherin, confirming the acquisition of a mesenchymal phenotype in both cell lines. E2 had no effect on the proliferation of both cell lines, at least for the treatment times we considered, either alone or in combination with Mix, unlike what was reported by other authors [40].

Our experimental findings on the role of E2 in cancer cell invasiveness are partially consistent with previous studies that underscore sex-based differences in lung cancer susceptibility and progression with a higher mortality rate among female patients with NSCLC compared to males [41,42]. Additionally, evidence of receptor-mediated tumor promotion by estrogens [43], as well as observed associations between hormone replacement therapy or oral contraceptive use and increased mortality [44], further support the relevance of hormonal factors in NSCLC outcomes.

Resistance to chemotherapy, particularly to platinum compounds such as CDDP, remains a major clinical challenge [45]. Our data show that pre-treatment with E2, Mix, or their combination reduced the sensitivity of NSCLC cells to CDDP, with the most pronounced resistance observed in the E2/Mix group. Similar results regarding the presence of E2 were obtained by Yu et al., who demonstrated that concentrations of E2 between 10 and 1000 nM cause a significant reduction in the cellular response to CDDP in H1650 NSCLC cells, compared to the use of cisplatin alone [7].

In NSCLC cells, E2 signaling has been shown to promote tumor progression through estrogen receptors (ERs) [1,2]. After demonstrating the presence of ERα and ERβ in both tumor cell lines and establishing that ERβ is present at higher levels, the use of the ER specific inhibitors MPP and PHTPP indicated that ERβ was directly involved in E2-induced tumor promotion. Estrogen treatment is known to activate both genomic and non-genomic signaling pathways via ERs [1,2]. In particular, E2 can rapidly induce the activation of membrane-associated ERs, leading to the transactivation of receptor tyrosine kinases, such as EGFR. Crosstalk between estrogen receptors and EGFR signaling represents a well-established mechanism by which estrogen amplifies kinase-driven pathways independently of direct ligand binding to EGFR. This results in the increased phosphorylation of EGFR and subsequent activation of downstream signaling cascades, including the PI3K/AKT and MAPK/ERK pathways [27,30].

In our experimental model, we find that E2 treatment not only increases the phosphorylation of EGFR but also appears to increase its expression, further amplifying downstream signaling. Different behaviors are observed for AKT and ERK. In A549 cells, an increase in the phosphorylation and transcription of both AKT and ERK is observed. By contrast, Calu1 cells show a reduction in the phosphorylated forms of the two proteins, while the unphosphorylated forms increase. Therefore, the mechanisms involved could differ between the two cell lines or have different activation and/or degradation kinetics. We hypothesize that this reflects the different oncogenic backgrounds of the two cell lines rather than a different response to E2. The two cell lines differ in their driver and tumor-suppressor genotypes, and both AKT and ERK are downstream nodes at which a constitutively active upstream input can saturate the pathway and mask a further receptor-driven increase in phosphorylation.

Estrogen signaling can promote NF-κB activation either directly or indirectly through AKT- and ERK-dependent mechanisms, contributing to the regulation of genes involved in cell survival, inflammation, and proliferation, consistent with the overall estrogen-induced signaling profile [46]. In our experimental model, we observed a much greater increase in E2-induced NF-κB in A549 cells than in Calu1 cells, although both cell lines exhibited an increase in cells with NF-κB-positive nuclei. These findings are consistent with previous studies showing that E2 activates interconnected signaling networks involving growth and survival pathways, such as MAPK/ERK, PI3K/AKT and NF-κB signaling. This occurs through both genomic and non-genomic mechanisms mediated by estrogen receptors [9,47,48].

Among the mechanisms identified as involved in drug resistance, autophagy, a key survival pathway, is known to be activated by cytokines and estrogens within the TME [49,50]. In our experiments, E2 and/or Mix treatments increased autophagic activity, as indicated by the LC3-II accumulation, p62 and YAP degradation [33,51], and these results point to canonical, degradative autophagy. The increased autophagic activity observed in NSCLC cell lines following the aforementioned treatments could therefore play multiple roles: (i) helping to maintain cellular homeostasis by eliminating dysfunctional organelles and macromolecules, thereby promoting cell survival in harsh conditions; (ii) increasing catabolism by contributing to the availability of building blocks for essential cellular processes, thus supporting the biosynthetic demands of rapidly proliferating tumor cells; and (iii) serving as a protective mechanism that contributes to the development of acquired drug resistance.

In addition to its well-known role in ATG-regulated protein degradation, recent reports have shown that the autophagic mechanism is also involved in paracrine signaling that regulates pro-tumor crosstalk between cancer cells and TME components [52]. The unconventional secretory pathway, known as secretory autophagy, provides cells with alternative release strategies for both normally secreted and stress-induced proteins, such as HMGB1 and inflammatory cytokines [53].

Secretory and degradative autophagy have been shown to be highly integrated, sharing several processes including initiation, nucleation, membrane expansion, autophagosome maturation and amphisome formation [35]. Recent research has revealed that crosstalk between exosome and autophagy mechanisms in response to stressful conditions induces fusion between autophagosomes and MVBs to form amphisomes, leading to the unconventional secretion of EVs [54,55].

The mechanisms that determine the switch from degradative to secretory autophagy remain incompletely understood. As reported by New and Thomas [18], the direction of autophagosome trafficking is influenced by membrane-associated proteins: syntaxin (STX17) directs toward lysosomal degradation, while the soluble N-ethylmaleimide sensitive factor attachment protein receptor (SNARE) SEC22B guides toward plasma membrane fusion. Starting from this model, we found that E2 promotes both degradative and secretory autophagy with a pronounced effect on the secretory pathway, especially when combined with pro-inflammatory cytokines. Interestingly, we also observed upregulation of SEC22B and its association with LC3-II, supporting the activation of secretory autophagy. This dual activation was confirmed by FRET analysis and TEM, which revealed vesicle formation near the plasma membrane in both of the cell lines evaluated.

Secretory autophagy has also been implicated in intercellular communication within the TME, facilitating the release of EVs that carry bioactive molecules [56]. Our data show that EVs isolated from the supernatants of tumor cells treated with E2 and Mix, alone or in combination, when administered to normal lung fibroblasts induced CAF markers (α-SMA, FAP, PDGFR-β, PDPN), confirming a functional role in fibroblast activation. These findings support a model in which E2 enhances pro-tumorigenic communication between tumor cells and fibroblasts via autophagy-mediated extracellular vesicle secretion. Estrogen regulation of EV secretion has been previously demonstrated by Drula et al. in ERα-positive breast cancer cells [57]. However, breast cancer is by nature prone to hormonal signaling, particularly estrogenic signaling. In non-small cell lung cancer cells, such as A549 and Calu1 cells, the regulation of EV production would instead occur through the activation of ERβ, which triggers the signaling cascade mediated by the EGFR/AKT/ERK biochemical pathway known to promote cell survival and tumor progression [58].

Further investigation is required to identify the EV-associated molecules responsible for fibroblast activation into CAFs and to elucidate the underlying mechanisms.

Estrogens in the TME originate not only from gonadal sources but may also be locally synthesized by tumor or stromal cells. Thus, systemic estrogen levels may not reflect the intratumoral concentrations that affect tumor biology. The effect of estrogens is also modulated by the relative abundance of ERs in different TME cell types. Both ERα and ERβ, as well as other receptors such as GPER and ERRs, are expressed in lung tissues. In our study, we focused on ERα and ERβ due to their prominent roles in NSCLC progression. Notably, ERβ expression is elevated in lung adenocarcinoma and correlates with poor differentiation and lymph node metastasis [59]. Our results showed that the use of PHTPP, a selective ERβ antagonist, but not MPP, a selective ERα antagonist, reduced E2-induced pro-tumor features in vitro, in line with previous reports [42,60]. ERβ has been observed to act as a tumor suppressor precisely by counteracting the pro-proliferative EGFR/AKT/ERK signaling pathways in mesothelioma [61]. However, in lung adenocarcinoma, ERβ expression has been associated with acquired resistance to EGFR-TKIs, through activation of the AKT/ERK pathway [62,63].

Overall, our findings suggest that estrogen signaling may contribute to a pro-tumor microenvironment through its influence on the crosstalk between tumor cells and CAFs.

A key strength of this study is its elucidation of how E2 facilitates tumor progression of NSCLC. By integrating in vitro experiments with computational analyses, we observed for the first time consistent patterns indicating that E2 promotes both secretory autophagy in tumor cells and the activation of CAFs, two processes known to facilitate tumor progression.

While confirmation of these data in patient-derived NSCLC cells would provide stronger evidence in terms of generalizability, the clinical relevance of this interaction is nevertheless supported by our observation that high expression of the MIX gene signature, comprising *TGFB1*, *IL-6*, *IL-8*, *CCXL-16* and *ESR2*, is associated with poorer overall survival in NSCLC patients. This molecular profile was also linked to elevated expression of secretory autophagy genes and CAF markers identified in our in vitro studies. Validation in three large clinical cohorts (TCGA-LUNG, OAK and POPLAR) reinforces the potential significance of this estrogen-related axis.

However, this study has one limitation that should be considered: the absence of in vivo experiments. Given the significant limitations of commonly available animal models of NSCLC, which do not fully reproduce the complexity of the human tumor microenvironment, our analysis, conducted on three independent public databases including more than 1500 patients, provides robust translational evidence linking our experimental findings with clinical relevance.

While our data do not directly address the broader TME, the consistent association between estrogen signaling and the tumor–CAF interaction points to a mechanism that may contribute to more aggressive tumor behavior and resistance to therapy. Deciphering the molecular and biochemical pathways activated by estrogen could reveal new therapeutic vulnerabilities, including potential sex-specific targets, to improve outcomes for patients with this still-fatal disease.

Our findings could support the development of combination strategies integrating estrogen-targeting agents with immunotherapy or molecularly targeted therapies, as has already been investigated in breast cancer [64] but is largely unexplored in lung cancer.

## 4. Materials and Methods

### 4.1. Cell Lines and Treatments

A549 (CCL-185) and Calu1 (HTB-54) cells were cultured in RPMI 1640 medium containing 10% charcoal stripped fetal bovine serum (FBS, Euro-Clone, Pero, Milan, Italy), L-glutamine, sodium pyruvate, and 100 units of penicillin–streptomycin in a humidified incubator at 37 °C with 5% CO_2_. Before each treatment, A549 (CCL-185) and Calu1 (HTB-54) cells were cultured for 16 h in RPMI 1640 medium (Thermo Fisher Scientific, Waltham, MA, USA) without FBS. Cells were treated for 24 h as follows: the mixed cytokines (Mix), estradiol (E2, Sigma-Aldrich Inc., St. Louis, MO, USA), or a combination of mixed cytokines and estradiol. Recombinant human TGF-β was obtained from R&D systems (Thermo Fisher Scientific), and IL-6, IL-8 and CCXL-16 were from PeproTech, Inc. (London, UK). The total concentration of the cytokine mixture used for the stimulation experiments was 10 ng/mL (2.5 ng/mL for each of the following cytokines: TGF-β, IL-6, IL-8 and CCXL-16); E2 was used at a concentration of 10 nM.

To evaluate apoptosis, cells treated as above were also challenged with cisplatin (CDDP, Sigma-Aldrich; 100 µM for A549 or 200 µM for Calu1) in the absence or presence of Mix, E2, or both. In addition, in one set of experiments, we treated cells with the selective ERβ antagonist 4-[2-phenyl-5,7-bis(trifluoromethyl)pyrazolo [1,5-a]pyrimidin-3-yl]phenol (PHTPP, Sigma-Aldrich, 10, 50 and 100 nM) or the ERα antagonist 1,3-bis(4-hydroxyphenyl)-4-methyl-5-[4-(2-piperidin-1-ylethoxy)phenol]-1H-pyrazole (MPP) starting 2 h before E2 administration, alone or in combination with Mix.

MRC5 cells (CCL-171), a fibroblast cell line derived from the normal lung tissue of a male donor, were used for the EV studies. The cells were cultured in Minimum Essential Medium (MEM; Thermo Fisher Scientific) containing 10% charcoal stripped FBS, L-glutamine, sodium pyruvate, and 100 units of penicillin–streptomycin in a humidified incubator at 37 °C with 5% CO_2_. MRC5 cells were incubated for 24 h with EVs isolated from a T75 flask of cells at approximately 75% confluence. The supernatant was collected for EV separation, and cells subjected to each treatment were counted. There were no significant differences in the number of cells counted between the different experimental conditions, consistent with the lack of cytotoxic and/or cytostatic effects observed with the different treatments.

In most of the published studies conducted in vitro, the cell culture conditions are not specified or whether media with added FBS is used is unknown, which can contain variable concentrations of estrogens, depending on the batch and the animal of origin. This makes the experimental conditions difficult to standardize, limiting the representativeness of the results obtained. On the contrary, in our experimental model, we worked in standardized and reproducible conditions by replacing the FBS with charcoal stripped fetal bovine, which is devoid of the possible presence of estrogens, and using a concentration of E2 (10 nM) considered as physiological. In our opinion, this aspect is very important for reproducing conditions as close as possible to those that occur in vivo or, in any case, to better evaluate the specific contribution of E2 to what is observed.

### 4.2. Migration Assay

Cell migration was examined by the scratch assay according to [65]. Cells were seeded into a 24-well plate. When the cells reached confluence, a cut was made on the plate with a sterile tip. After washing with phosphate-buffered saline (PBS), the cells were imaged at T0 and then treated, in medium without FBS, with E2 (10 nM) with or without 10 ng/mL Mix. The cut region was monitored for 48 h, and images (10 per well) were acquired through a digital camera system coupled with an inverted microscope (IX-71 Olympus Corporation, Tokyo, Japan). Cell migration across the region was analyzed and quantified using ImageJ v1.48 software.

### 4.3. Invasion Assay

Cell invasion was analyzed by 8-micron-pore-size polycarbonate membrane Matrigel-coated transwell inserts (Corning Inc., Corning, NY, USA). A549 and Calu1 cells were sown inside the chamber in 0.5 mL of culture medium without FBS, while culture medium with FBS was placed underneath. The cells were maintained at 37 °C in a humidified atmosphere containing 5% CO2 for 48 h. After removing the cells on the top with a cotton swab, the membranes were washed with PBS and fixed with 4% paraformaldehyde and stained with Hoechst 33258 (Sigma-Aldrich Inc., St. Louis, MO, USA). Through a fluorescence microscope, both the cells that passed through the filter and the cells adhered to the bottom of the well were counted. With both measurements carried out, comparable results were obtained. All experiments were performed in triplicate. At least five fields were counted in each experiment.

### 4.4. Cell Viability Assay

Cell viability in response to E2 and cytokine Mix treatments was evaluated by the CellTiter-GLO luminescent assay (Promega Corporation, Madison, WI, USA) according to the manufacturer’s protocol. Briefly, A549 and Calu1 cells were plated at 5000 cells/well in 96-well flat-bottom plates and incubated overnight. Before drug treatments (for 24 h and 48 h), the cells were serum-starved for 8 h. The assay was performed twice in quintuplicate.

### 4.5. Fluorescence Microscopy

Control and treated cells were fixed with 4% paraformaldehyde, permeabilized with 0.5% Triton X-100, and incubated for 1 h at 4 °C with the following antibodies: SEC22B (Thermo Fisher Scientific, MA, USA), LC3 and α-SMA (Novus Biologicals, Littleton, CO, USA), SQSTM1/p62 (Sigma-Aldrich), and NF-κB (Santa Cruz Biotechnology Inc., Dallas, TX, USA). As secondary antibodies, anti-mouse IgG AlexaFluor 488-conjugated antibodies and anti-mouse IgG AlexaFluor 594-conjugated antibodies (both Invitrogen) were used. For analysis of actin stress fibers, we used anti-phalloidin TRITC-conjugated antibodies (Sigma-Aldrich).

After washing with PBS, the cells were counterstained with Hoechst 33258 and finally mounted in fluorescence mounting medium (Dako, Glostrup, Denmark). Images were acquired with intensified video microscopy (IVM) with an Olympus fluorescence microscope (Olympus Corporation, Milan, Italy) equipped with a CoolLed pE-300-W (CoolLED Ltd., Andover, United Kingdom).

Morphometric analysis of NF-κB was performed on at least 50 cells for each experimental sample, with three different investigators counting NF-κB-positive nuclei blindly and independently.

### 4.6. Western Blot

Cells were lysed in RIPA lysis buffer with added protease and phosphatase inhibitors. The amount of protein loaded was determined using Varioskan Lux (Thermo Scientific). The samples were subsequently loaded onto a polyacrylamide gel (Invitrogen, Carlsbad, CA, USA), electrotransferred onto PVDF membranes and incubated with the following antibodies: E-Cadherin, Vimentin, YAP, PDGFR- β, EGFR, NFkB, ERK 1/2, AKT (all Santa Cruz), FAP (Thermo Fisher Scientific), LC3 (Novus Biologicals), p62 (Sigma-Aldrich), p-EGFR (Cell Signaling Technology, Danvers, MA, USA), p-ERK 1/2 and p-AKT (BD Transduction Laboratories, San Jose, CA, USA), α-SMA (Novus Biologicals), Podoplanin (PDPN, Cell Signaling) Alix (Thermo Scientific), TSG101 (GeneTex), and HSP90α/β (Abcam). After the membranes were washed, immune complexes were detected with horseradish peroxidase-conjugated species-specific secondary antibodies (Jackson Laboratory, Bar Harbor, ME, USA). Membranes were developed using ECL detection reagents (Millipore Corporation, Billerica, MA, USA). Reactive bands were detected with the ChemiDocMP system (Bio-Rad Laboratories Inc., Hercules, CA, USA). To ensure the presence of equal amounts of protein, the membranes were again probed with anti-GAPDH or HSP70 (all Santa Cruz). Protein expression levels were quantified with densitometric analysis using NIH ImageJ v1.48 software.

### 4.7. Flow Cytometry

#### 4.7.1. Cell Viability

Control and treated cells were incubated with 1 μm Calcein-AM (Thermo Fisher Scientific) at 37 °C for 30 min. In live cells, non-fluorescent Calcein-AM is converted to a green fluorescent dye (green-fluorescence-positive cells), whereas dead cells with compromised cell membranes do not retain Calcein and thus do not display green fluorescence (green-fluorescence-negative cells).

#### 4.7.2. Apoptosis

Control and treated cells were stained with a fluorescein isothiocyanate (FITC)-conjugated annexin V (AV) and propidium iodide (PI) detection kit (Marine Biological Laboratory, Woods Hole, MA, USA). The test allows us to discriminate early apoptosis (AV/PI negative) from late apoptosis (double positive AV/PI) or necrosis (single PI positive).

#### 4.7.3. Actin Microfilament Polymerization

Quantification of filamentous actin (F-actin), globular actin (G-actin) and total (F + G) actin was analyzed to assess changes in the microfilament system of the cytoskeleton. Cells were fixed with 4% paraformaldehyde, permeabilized with 0.5% Triton X-100 and incubated for 45 min at 37 °C with DNase I (Invitrogen) for G-actin detection and phalloidin (Sigma) for F-actin detection. For total actin detection, cells were incubated with monoclonal antibody against actin (Santa Cruz), followed by AlexaFluor 488-conjugated anti-mouse IgG (Invitrogen). The median fluorescence intensity values were used for semiquantitative assessment of F-actin, G-actin and total actin.

#### 4.7.4. Fluorescence Resonance Energy Transfer (FRET)

We applied FRET analysis with flow cytometry to study the molecular association of LC3 and SEC22B in cells that were untreated (CTR) or treated with Mix, E2 or a combination of Mix/E2. Briefly, the cells were fixed and permeabilized, as described above, for immunofluorescence and labeled with antibodies tagged with donor (PE) or acceptor (Cy5) dyes. The following primary and secondary antibodies were used: LC3 (Santa Cruz Biotechnology), anti-SEC22B (Thermo Fisher Scientific), PE-labeled anti-mouse (Sigma-Aldrich) and Cy5-labeled anti-rabbit (AbCam, Cambridge, MA, USA). YAP protein was detected in the FL2 channel (PE, donor), TBC1D2 in FL4 (Cy5, acceptor) and FRET in the FL3 channel. Quantification of protein–protein interactions was obtained by calculating the FRET efficiency (FE) by using the following Riemann algorithm [66]: FE = [FL3DA − FL2DA/a − FL4DA/b]/FL3DA, in which A is the acceptor and D the donor, and where a = FL2D/FL3D and b = FL4A/FL3A.

### 4.8. Isolation and Characterization of Small Extracellular Vesicles

EVs were isolated from A549 and Calu1 cell conditioned medium previously supplemented with 10% FBS exosome-depleted medium (Thermo Fisher Scientific). For the isolation of EVs, the culture supernatants (12 mL per sample) of treated and untreated cells were subjected to sequential centrifugations.

The first one was at 300× *g* for 10 min. The culture supernatants were centrifuged at 2000× *g* for 20 min at 4 °C to remove cell debris and then ultracentrifuged with an SW41Ti rotor (Beckman Coulter, Brea, CA, USA) at 10,000× *g* for 20 min to eliminate macrovesicles and various aggregates. EVs containing exosomes were isolated by ultracentrifugation at 100,000× *g* for 3 h. The pellet was washed with PBS at 100,000× *g* for 3 h. The concentration and particle size distribution of purified EVs were tracked by nanoparticle tracking analysis (NTA) using a Nanosight NS300 system (Nanosight technology, Malvern, UK) configured with a 488 nm laser and a high-sensitivity scientific complementary metal-oxide semiconductor (sCMOS) camera. For each sample, multiple videos of 60 s duration were recorded, generating replicate histograms that were averaged. The videos have been analyzed by the in-build NanoSight Software NTA 3.4.4.

### 4.9. Transmission Electron Microscopy

Control and treated cells were fixed with 2.5% glutaraldehyde in 0.1 M sodium cacodylate buffer (pH 7.2) for 1 h at room temperature. After washing, the cells were post-fixed in 1% osmium tetroxide (OsO4) in 0.1 M sodium cacodylate buffer for 1 h at room temperature. The samples were then washed in the same buffer and dehydrated through serial ethanol solutions (30–100%) and embedded in an epoxy resin. Using a UC6 ultramicrotome (Leica, Wetzlar, Germany), ultrathin sections (60 nm) were made, subsequently stained with uranyl acetate and then contrasted with lead hydroxide and examined at 100 kV with a Philips EM 208S transmission electron microscope (FEI-Thermo Fisher) equipped with a Megaview III SIS camera system (Olympus-SIS, Milan, Italy) and iTEM3 software (v8.3.x.).

Morphometric analysis was performed considering 10 cells for each experimental sample in which three different investigators, blindly and independently, counted secretory vesicles, autophagic vacuoles, and multivesicular bodies. The results are reported as the mean ± SD of the counts performed by the three investigators.

### 4.10. Computational Analysis of Public Data on Estrogen’s Role in Secretory Autophagy and CAF Activation

#### 4.10.1. Gene Expression Analysis and Estrogen Receptor Proxy Selection

Normalized gene expression data (TPM) from the TCGA-LUNG cohort was used for analysis. Patients with complete *estrogen receptor 1* (*ESR1*) and *estrogen receptor 2* (*ESR2*) gene expression data and survival information were included in the analysis. Given that smoking represents the predominant risk factor for NSCLC and a potential confounding factor, the TCGA-LUNG cohort used for survival analyses was further restricted to patients with a documented smoking history (excluding lifelong non-smokers, based on the TCGA clinical annotation). To identify a suitable proxy for E2 expression, the expression levels of *ESR1/2* were compared between male and female patients within the cohort. Statistical significance for expression differences was assessed using the Wilcoxon rank-sum test to account for the potential non-normal distribution of gene expression data. Based on these comparisons, *ESR2* was selected as the proxy for downstream analyses, enabling the inclusion of both male and female specimens.

#### 4.10.2. Gene Signature Construction and Patient Stratification

A composite gene signature, termed “MIX”, was constructed using the expression levels of genes corresponding to the cytokines used within the Mix (TGFB1, IL-8, IL-6, CCXL-16), along with ESR2 gene expression. For each patient in the TCGA-LUNG, OAK, and POPLAR cohorts, a “MIX score” was calculated through the GSVA R package using the “ssgsea” method. Patients within each cohort were then stratified into two groups: MIX-HIGH and MIX-LOW based on the median expression value of the calculated MIX score for each respective dataset. Patients with MIX scores above the median were classified as MIX-HIGH, and those below were classified as MIX-LOW.

#### 4.10.3. Survival Analysis

Kaplan–Meier survival curves for OS were generated for the MIX-HIGH and MIX-LOW patient groups within each cohort. Differences in OS between the groups were evaluated using the log-rank test. As a sensitivity analysis, this survival stratification was additionally repeated using ESR2 expression alone and using the four cytokine genes alone (TGFB1, CXCL8, IL6, and CXCL16, excluding ESR2) to determine whether the prognostic association depended on any single component of the MIX signature (Supplementary Figure S7, Supplementary Table S1). Moreover, to assess histologic subtype (lung adenocarcinoma, LUAD, vs. squamous cell carcinoma, LUSC) as a potential confounder, a Cox proportional-hazards model including a MIX-HIGH/LOW × histology interaction term and an additive model adjusting for histology as a covariate were fitted to the TCGA-LUNG cohort. Differential expression of ESR2 and the four CAF activation markers between the MIX-HIGH and MIX-LOW groups was similarly re-evaluated using linear models incorporating a strata × histology interaction term (Supplementary Figures S8 and S9, Supplementary Tables S2 and S3). The analysis was performed with the survival and survminer R packages.

#### 4.10.4. Secretory Autophagy Gene Signature Scoring

To build a secretory autophagy signature, we used the ATG7, ATG9A and MAP1LC3B genes, which are established master regulators of the autophagic machinery, along with the TRIM16, RAB8A and SEC22B genes, known to be involved specifically in the secretory autophagy process. The signature score was calculated for each patient using the GSVA R package choosing “ssgsea” as the method for the calculation. Differences in secretory autophagy signature scores between the MIX-HIGH and MIX-LOW groups were assessed using the Wilcoxon rank-sum test.

#### 4.10.5. Fibroblast Activation Marker Expression Analysis

The expression levels of four known CAF activation markers (ACTA2, FAP, PDGFR-β and PDPN) were analyzed across the MIX-HIGH and MIX-LOW groups in all cohorts. Gene expression values for these markers were extracted from each dataset. Statistical comparisons of expression levels between the two groups were performed using the Wilcoxon rank-sum test.

### 4.11. Data Analysis and Statistics

Data were analyzed using Student’s two-tailed *t*-test or ANOVA and post hoc *t*-test with Bonferroni correction for multiple comparisons (Prism v9, GraphPad Software Inc., San Diego, CA, USA). To determine if variables followed a normal distribution Shapiro–Wilk’s test was used. Homoscedasticity was assessed by Bartlett’s test or the F-test. For flow cytometry experiments, samples were acquired with a FACScalibur cytometer (BD Biosciences Inc., San Diego, CA, USA) equipped with a 488 nm Argon laser and with a 635 nm red diode laser and were analyzed using CellQuest software (v5.x, BD Biosciences). At least 20,000 events were acquired for each sample. All the statistical analyses performed on publicly available transcriptomics datasets were carried out using the R statistical environment (v4.4.1). All Benjamini–Hochberg adjusted *p*-values < 0.05 were considered statistically significant.

All data reported in this paper were verified in at least three independent experiments and are reported as the mean ± standard deviation (SD). Statistical significance was set at *p* < 0.05. In the figures, * *p* < 0.05; ** *p* < 0.01, *** *p* < 0.001, and **** *p* < 0.0001.

## 5. Conclusions

The experimental data reported here indicate that E2, alone or in combination with a mix of inflammatory cytokines, activates NSCLC cell survival pathways promoting the acquisition of malignancy-associated characteristics. Furthermore, by favoring secretory autophagy, it triggers an aberrant interaction between tumor cells and CAFs mediated by EVs, thereby contributing significantly to the progression and spread of the tumor.

We recognize that CAFs are not the only or the most important players in the TME. Indeed, it is highly complex, and the immune cells present therein, including tumor-associated macrophages (TAMs), are in constant dialog with CAFs and tumor cells, contributing to a tumor-promoting or tumor-unfavorable environment. Furthermore, our conclusions, based on observations in established cell lines, would need to be confirmed in patient-derived models.

However, in line with and supporting our experimental data, validating these results in the clinical setting lends considerable importance and translational relevance to these findings.

Indeed, the analysis of two large clinical cohorts identifies E2-modulated secretory autophagy as an emerging target for the development of personalized treatments and the design of novel combination therapies for NSCLC.

## Data Availability

TCGA-LUNG: RNA sequencing (RNA-Seq) gene expression profiles and associated clinical data for lung adenocarcinoma (ADC) and lung squamous cell carcinoma (SCC) patients were downloaded from the TCGA database via the UCSC Xena platform (https://xenabrowser.net/). OAK and POPLAR: Pre-immunotherapy treatment RNA-Seq profiles from the OAK (*n* = 700, phase 2) and POPLAR (*n* = 193, phase 3) clinical trials were obtained after request from European Genome-phenome Archive (EGA, https://ega-archive.org/) under the study accession number EGAS00001005013.

## Supplementary Materials

The following supporting information can be downloaded at: https://www.mdpi.com/article/10.3390/ijms27167490/s1.

## Abbreviations

The following abbreviations are used in this manuscript:

NSCLC	Non-small cell lung cancer
ER	Estrogen receptor
ERα	Estrogen receptor alpha
ERβ	Estrogen receptor beta
E2	17-Beta-estradiol
EVs	Extracellular vesicles
TME	Tumor microenvironment
TGF-β	Transforming growth factor beta
IL-6	Interleukin 6
IL-8	Interleukin 8
CCXL-16	(C-X-C Motif) ligand 16 chemokine
PHTPP	4-[2-Phenyl-5,7-bis(trifluoromethyl)pyrazolo [1,5-a]pyrimidin-3-yl]phenol
MPP	1,3-Bis(4-hydroxyphenyl)-4-methyl-5-[4-(2-piperidin-1-ylethoxy)phenol]-1H-pyrazole
EMT	Epithelial–mesenchymal transition process
CDDP	Cisplatin
CAFs	Cancer-associated fibroblasts
FAP	Fibroblast-activated protein
PDGFR-β	Platelet-derived growth factor receptor-beta
PDPN	Podoplanin
α-SMA	Alpha smooth muscle actin
EGFR	Epidermal growth factor receptor
NFkB	Nuclear factor kappa-light-chain-enhancer of activated B cells
ERK	Extracellular signal-regulated kinase
