# Supplementary material for: 17β-Estradiol Modulates Cancer Cell–Fibroblast Communication via Autophagy-Mediated Extracellular Vesicle Secretion and Promotes Poor Prognosis in Non-Small Cell Lung Cancer
Source: Int J Mol Sci. 2026 Aug 21;27(16):7490. doi: 10.3390/ijms27167490 (PMC13513166; doi:10.3390/ijms27167490)
Supplement: Supplementary file 1 [file ijms-27-07490-s001.zip › Revised supplementary figures final.pdf]

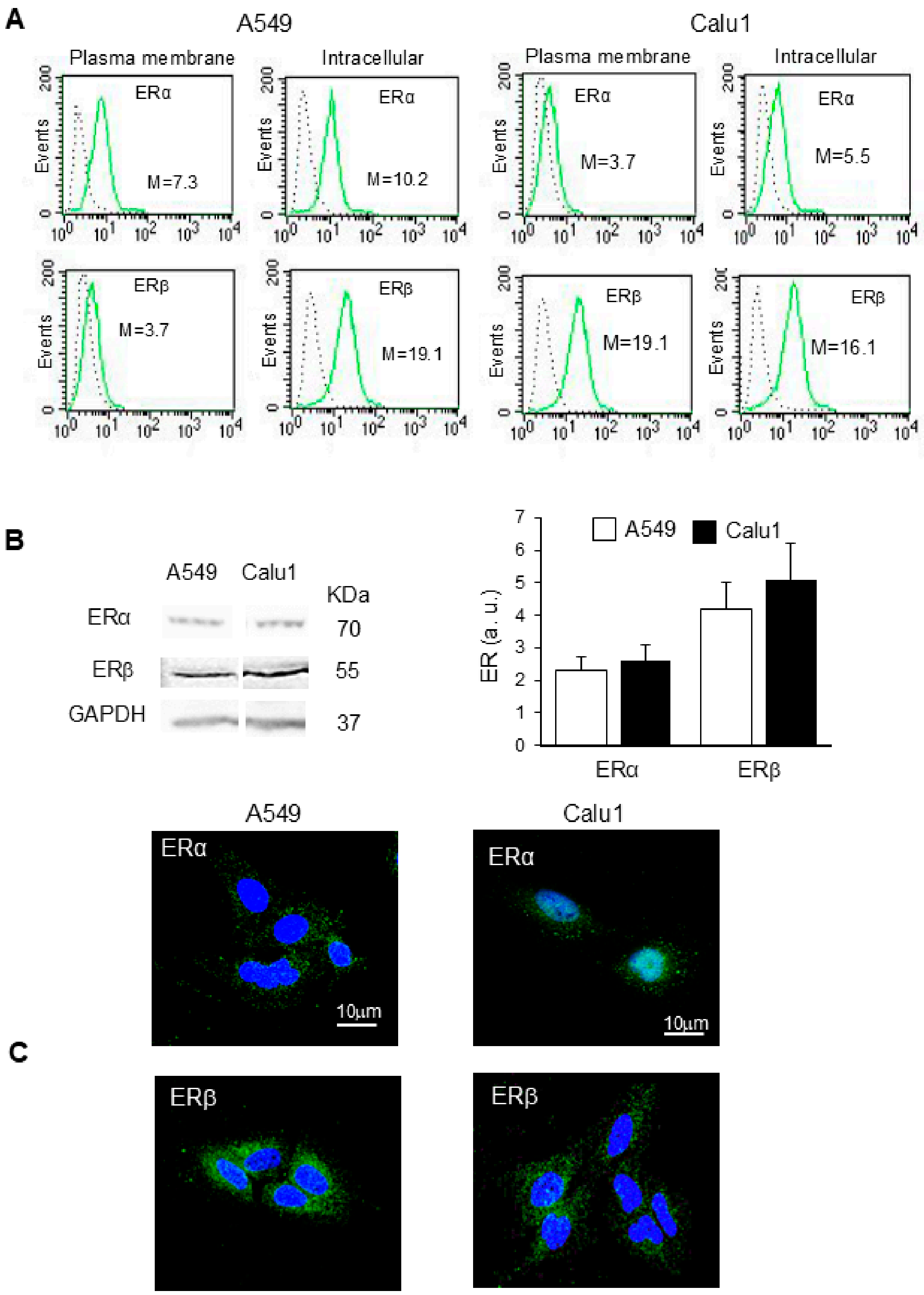

Supplementary Figure S1. ***ERα and ERβ expression in A549 and Calu1 cells.*** (**A**) Representative flow cytometry histograms showing ERα and ERβ expression on the plasma membrane or inside the cell in A549 (left panels) or Calu1 (right panels), as indicated. (**B**) Western blot analysis of ERα and ERβ in untreated A549 and Calu1 cells. Bar graph shows densitometric analyses of each protein normalized to GAPDH used as loading control. Data are expressed as the mean ± SD of three independent experiments. (**C**) Immunofluorescence micrographs showing representative images of A549 (left panels) and Calu1 (right panels) cells stained for ERα or ERβ (green) and counterstained with Hoechst (blue).

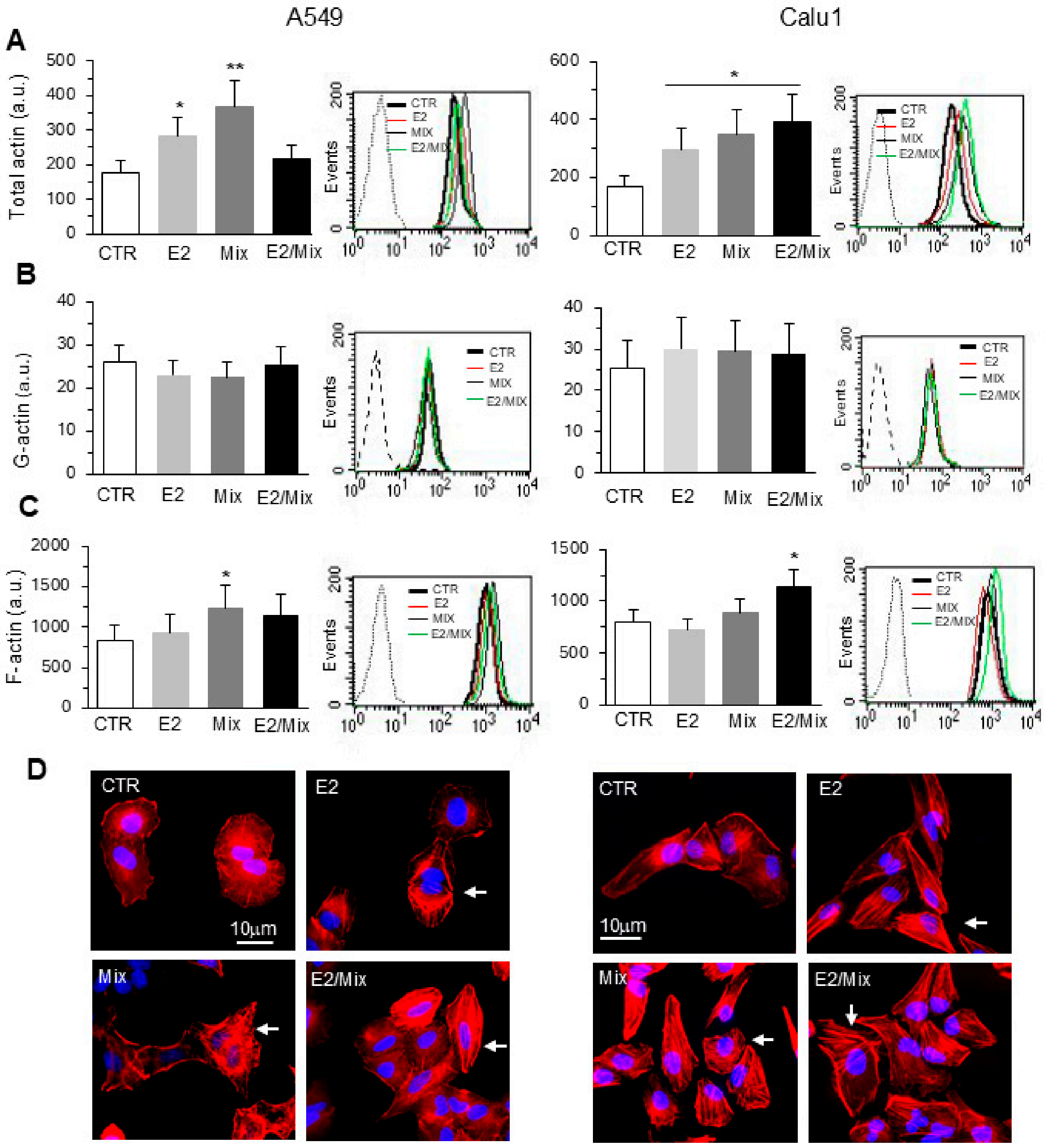

Supplementary Figure S2. ***E2 induces actin polymerization.*** Left panels: A549 cells and right panels: Calu1 cells. Histograms showing flow cytometry analysis obtained in a representative experiment of actin polymerization state performed in cells stained with (**A**) anti-actin antibody for total actin detection (**B**) DNAsi for G-actin evaluation, or (**C**) with Phalloidin for F-actin determination. Bar graphs report results obtained in three independent experiments and reported as mean ± SD. *$p < 0.05$ *vs*. CTR. (**D**) Representative fluorescence microscopy micrographs of control and treated cells stained with Phalloidin-TRITC (red) and counterstained with Hoechst. Arrows indicate stress fibers formation.

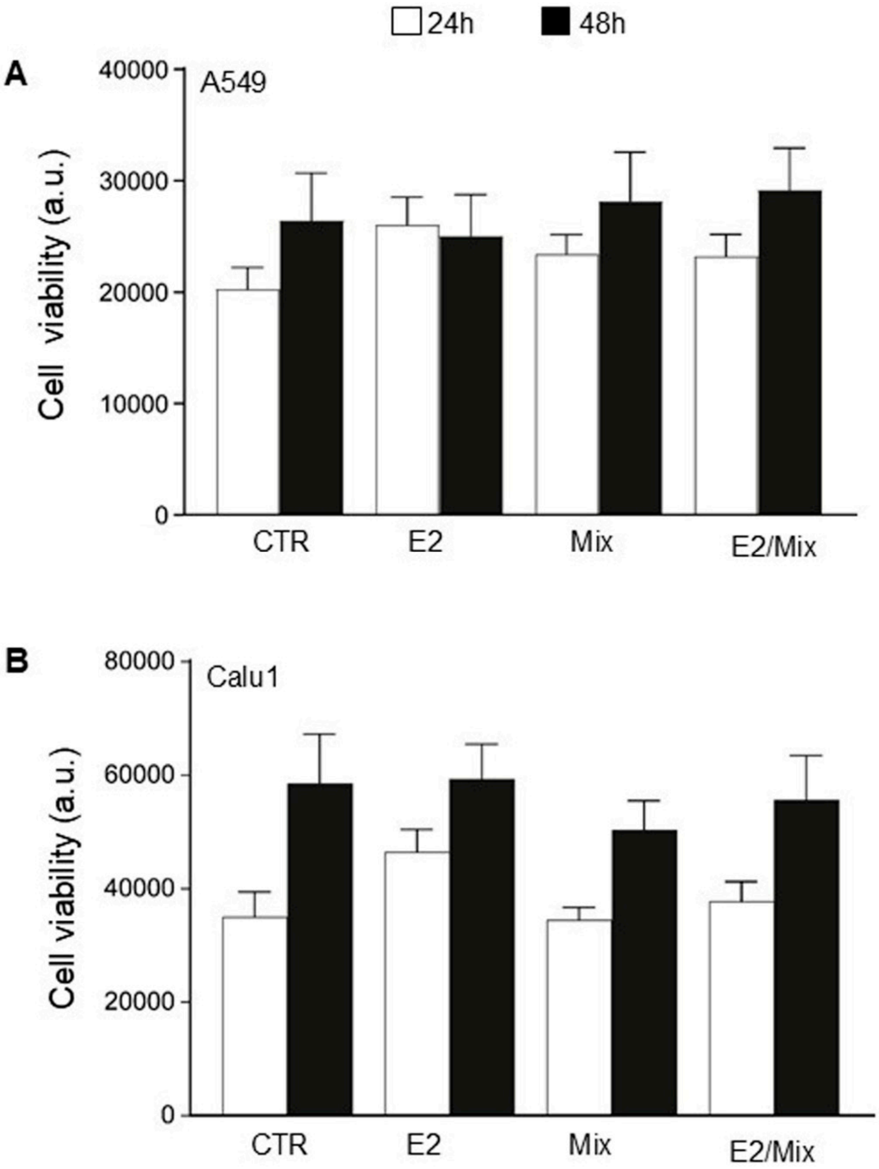

Supplementary Figure S3. ***E2 does not affect proliferation of either A549 or Calu1 cells.*** Bar graphs show cell viability evaluated by CellTiter-GLO luminescente assay after 24 and 48 h in (**A**) A549 or (**B**) Calu1 cells left untreated (CTR) or treated with E2, Mix, or E2/Mix. The results shown are the mean ± SD of experiments carried out twice in quintuplicate.

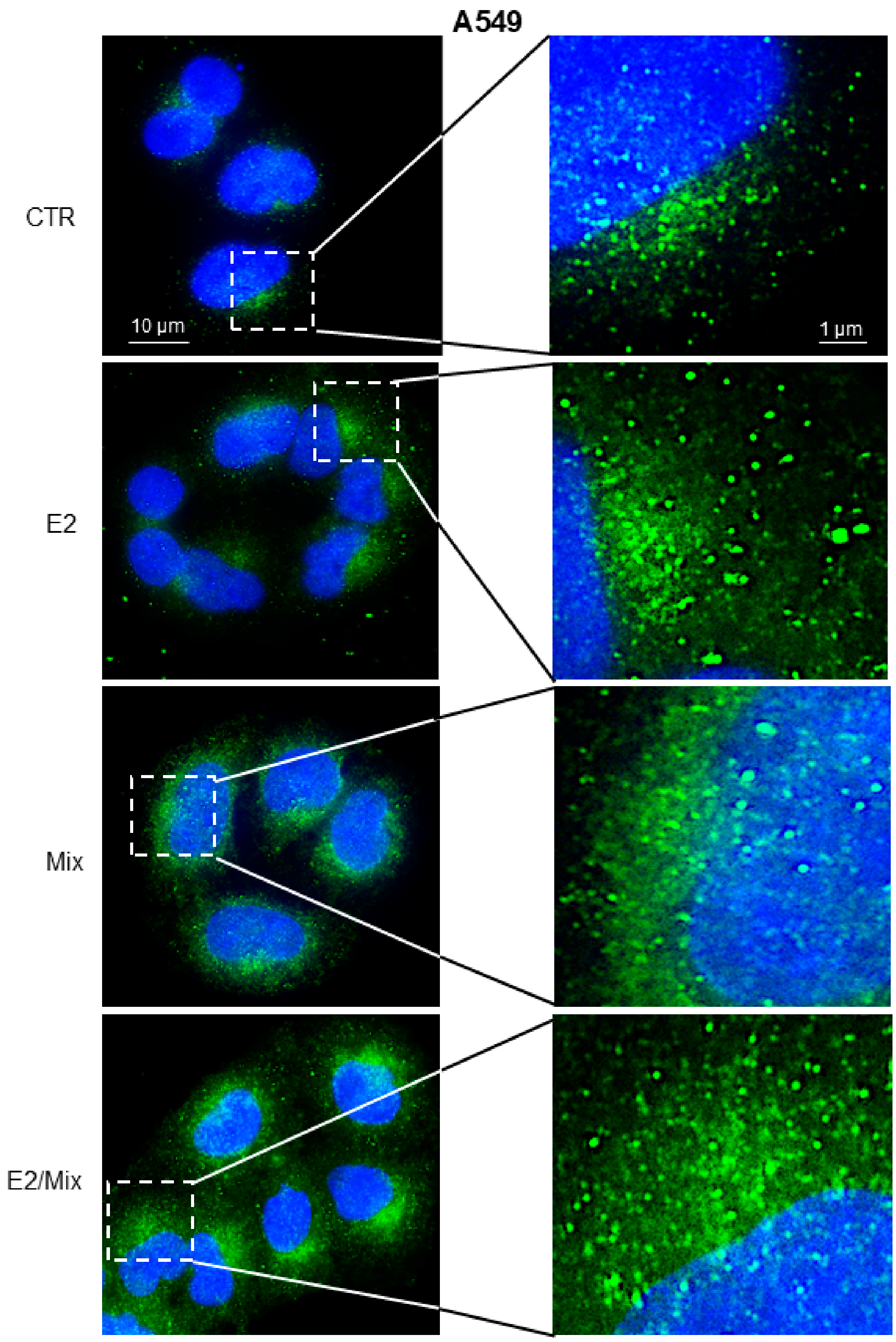

Supplementary Figure S4. *Left column.* Representative fluorescence microscopy micrographs of A549 cells untreated (CTR) or treated as indicated stained with anti-LC3 as and counterstained with Hoechst. *Right column.* Endogenous LC3-II is visualized as cytoplasmatic puncta representing autophagosomes, highly visible autophagosomes at higher magnification.

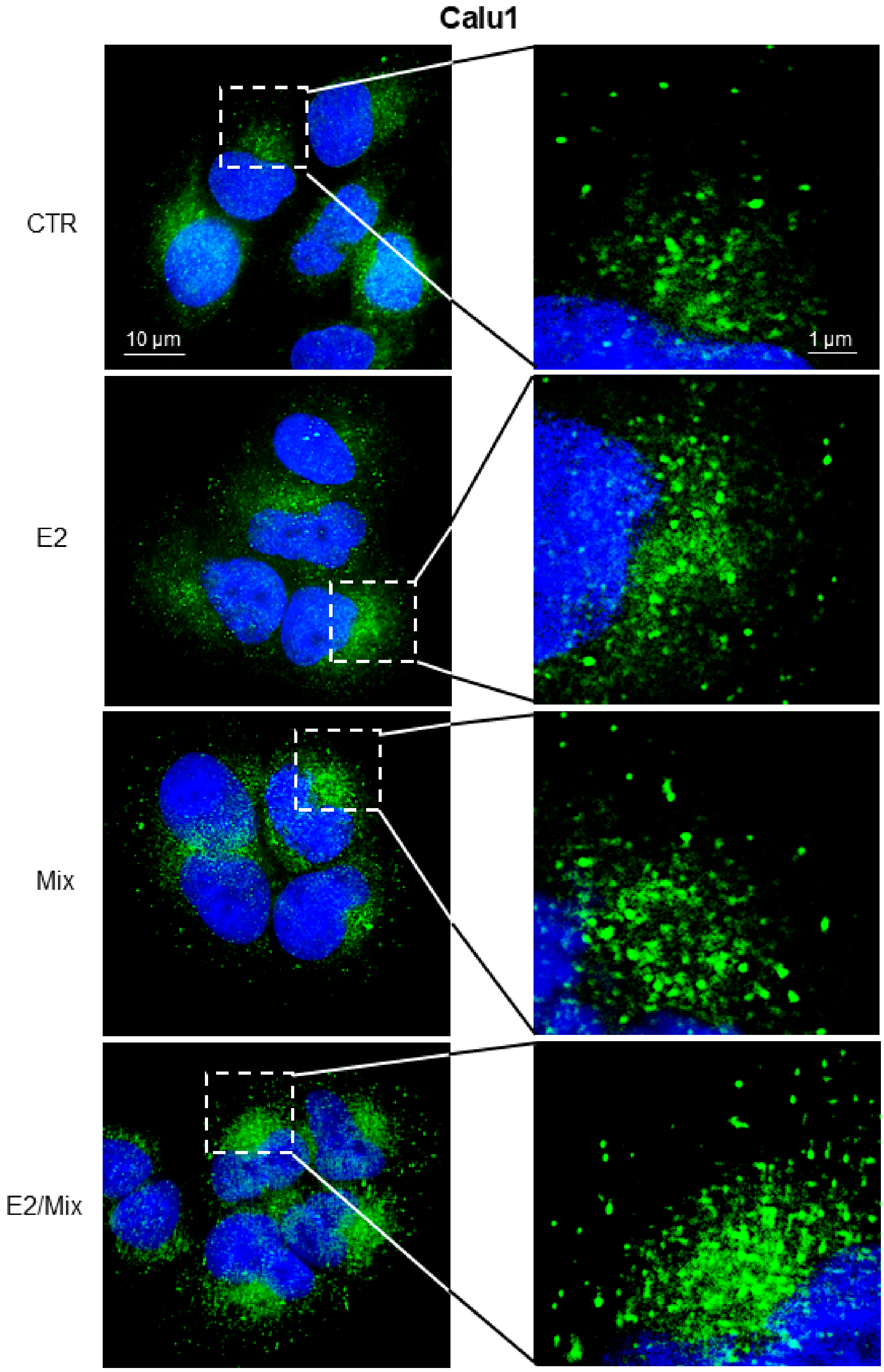

Supplementary Figure S5. *Left column.* Representative fluorescence microscopy micrographs of Calu1 cells untreated (CTR) or treated as indicated stained with anti-LC3 as and counterstained with Hoechst. *Right column.* Endogenous LC3-II is visualized as cytoplasmatic puncta representing autophagosomes, highly visible autophagosomes at higher magnification.

A

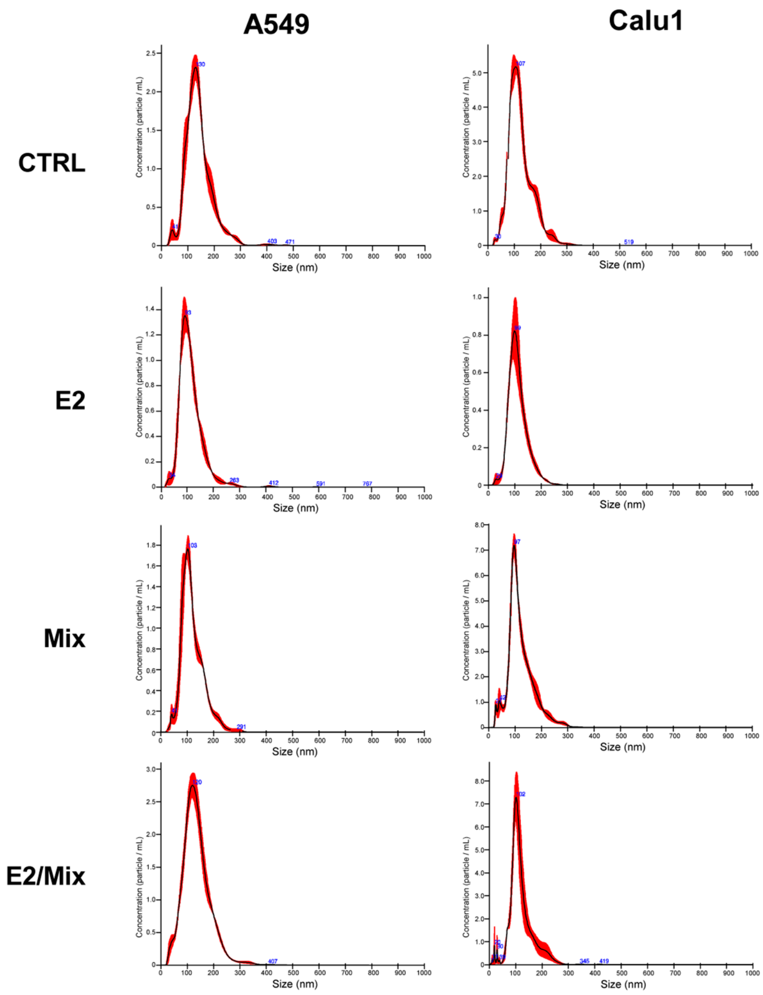

B

	A549				Calu1			
	CTRL	E2	Mix	E2/Mix	CTRL	E2	Mix	E2/Mix
**Diameter** (nm; mean ± SE)	141.6±4.6	115.2±1.9	120.3±5.2	135.1±0.7	125.1±0.7	110.5±1.3	120.7±0.8	116.7±8.4
**Tot. particle number** (billion)	2.15	1.90	2.02	2.30	4.38	5.73	5.30	4.24

C

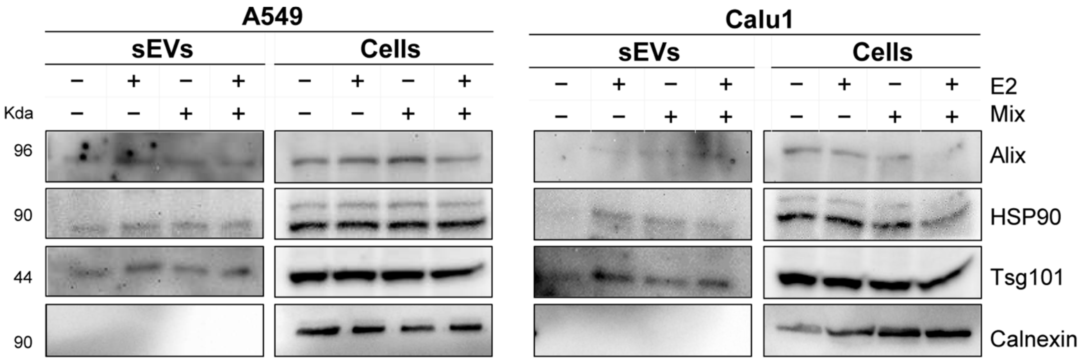

Supplementary Figure S6. Characterization of EVs derived from A549 and Calu1 cells. (A) NTA analysis was performed on EVs isolated by sequential ultracentrifugation from the supernatants of A549 and Calu1 cells either untreated (CTR) or treated with E2, Mix, or E2/Mix. A representative curve showing

vesicle size distribution (nm) is shown. Each curve was generated from three measurements per sample, and an average value was reported. (B) The table presents the dimensions and the total number of isolated particles for each sample under the specified conditions, as analyzed by NTA. The vesicles were homogeneous in size with a mean of 110-140 nm, which is the size range commonly attributed to EVs. The relative number of particles concentration of vesicles secreted from A549 and Calu1 cells did not change significantly before and after several treatments. (C) The protein expression of EVs markers was measured by Western blotting. Tsg101, Alix and Hsp90 were assayed as EVs positive markers while Calnexin was used as a negative marker to exclude contaminating apoptotic bodies or free protein. Total cellular extracts (cells) are shown as a control (right panel). The blot images are representative of three independent experiments.

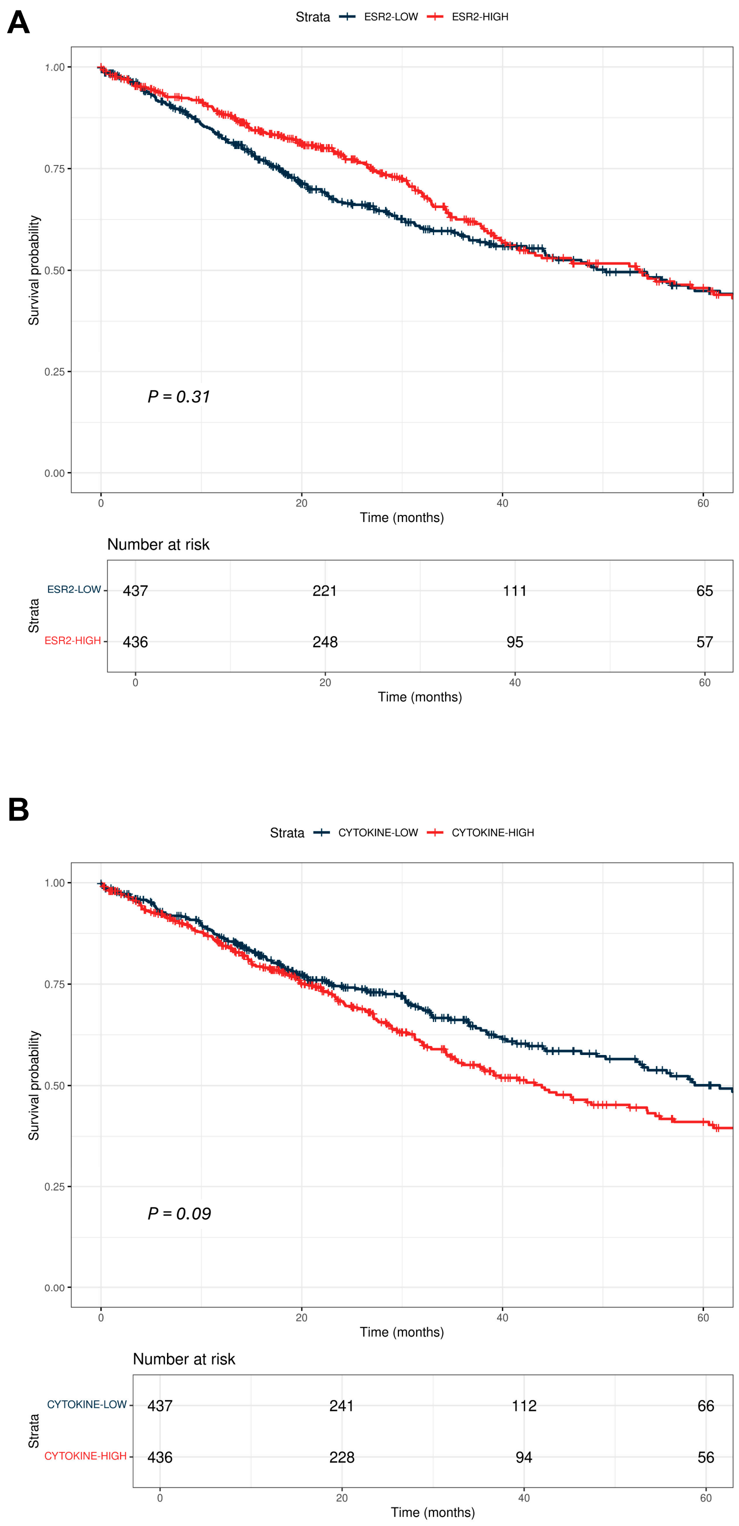

Supplementary Figure S7. ESR2 circularity sensitivity analysis in TCGA-LUNG. Kaplan-Meier curves showing overall survival (OS) for TCGA-LUNG cohort patients (same smoker-restricted population as Figure 10B) stratified by median expression into HIGH and LOW groups based on (**A**) ESR2 expression alone, and (**B**) the score of the four inflammatory cytokines alone (TGFB1, CXCL8, IL6, CXCL16; ESR2 excluded). Neither stratification reached statistical significance individually (p = 0.312 and p = 0.090, respectively, log-rank test).

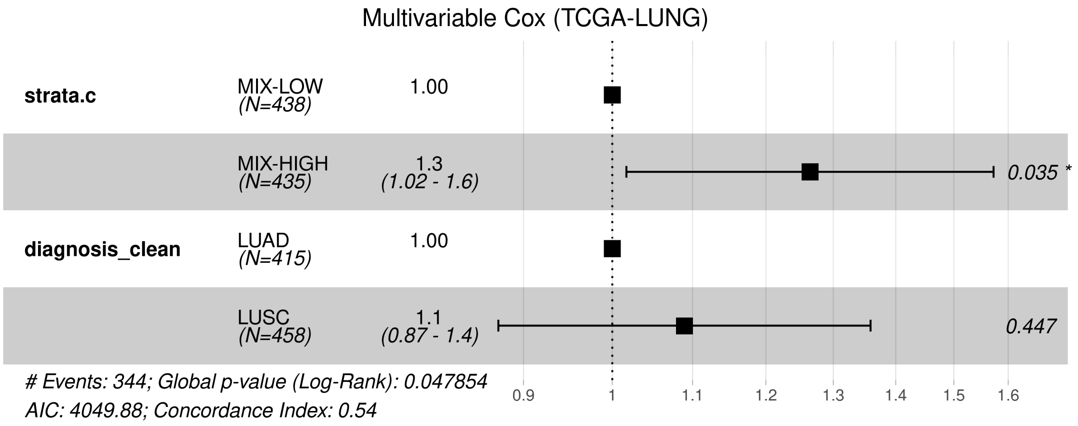

Supplementary Figure S8. Histologic subtype does not confound the MIX-HIGH/LOW survival association in TCGA-LUNG. Forest plot of a multivariable Cox proportional- hazards model for overall survival, including MIX-HIGH/LOW status and histologic subtype (LUAD vs. LUSC) as covariates. The MIX-HIGH/LOW effect remains significant after adjustment for histology (HR = 1.26, 95% CI 1.02–1.57, p = 0.035), while histologic subtype is not independently prognostic in this model (HR = 1.09, p = 0.447). A separate Cox model including a MIX-HIGH/LOW × histology interaction term showed no evidence that the prognostic effect differs between the two histologies (p = 0.589, **Supplementary Table 2**).

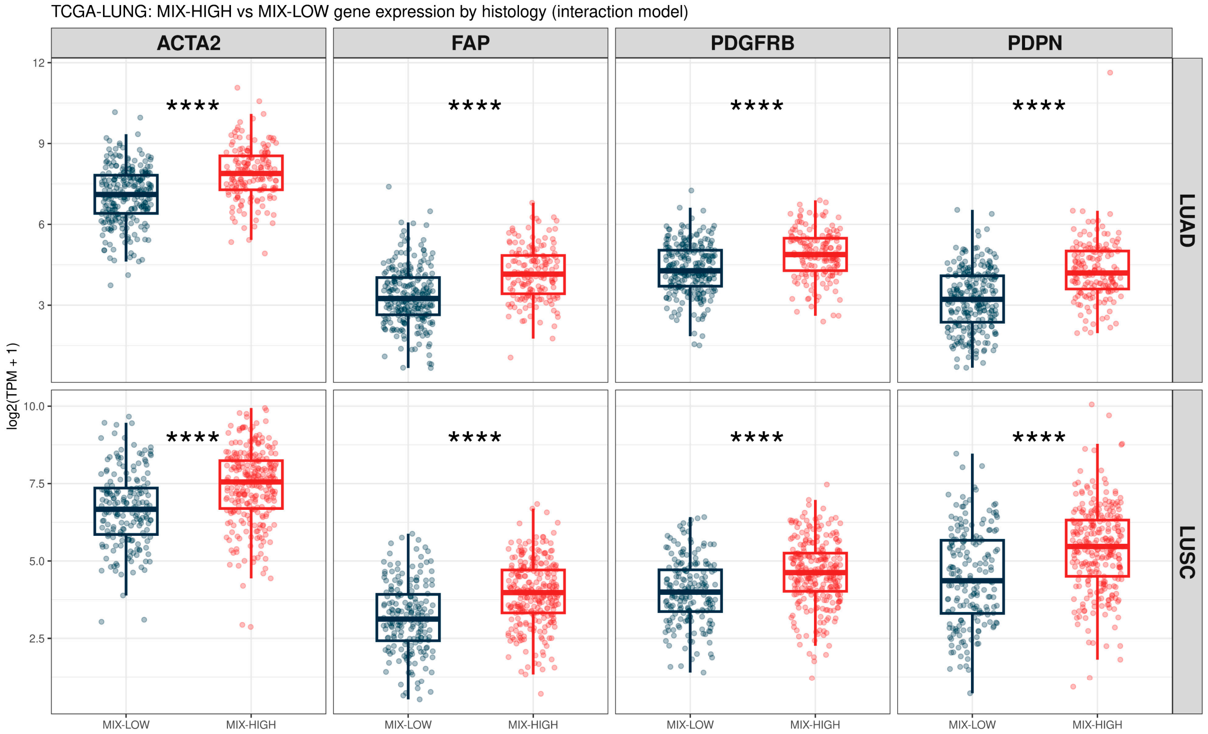

Supplementary Figure S9. Histologic subtype does not modify the MIX-HIGH/LOW difference in CAF-marker expression in TCGA-LUNG. Boxplots showing log_2(TPM+1) expression of ACTA2, FAP, PDGFRB and PDPN in MIX-HIGH versus MIX-LOW patients, stratified by histologic subtype (LUAD, top row; LUSC, bottom row; Wilcoxon rank-sum test within each panel). For all four genes, the MIX-HIGH/LOW difference is significant within both histologic subtypes. A linear model with a MIX-HIGH/LOW × histology interaction term confirmed that the magnitude of this difference does not depend on histologic subtype for any of the four genes (interaction $p > 0.58$ in all cases; **Supplementary Table 3**). Equivalent results for ESR2 (within-LUAD effect $p = 0.177$; interaction $p = 0.344$) are provided in the source data table but not plotted. For boxplots, the center line indicates the median, the box represents the interquartile range (IQR), and whiskers extend to 1.5 times the IQR from the box edges. *, **, ***, and **** denote $p < 0.05$, $p < 0.01$, $p < 0.001$, and $p < 0.0001$, respectively.
